# A bioadhesive antioxidant dual-crosslinked hydrogel mitigates endothelial pyroptosis to enhance ischemic flap survival

**DOI:** 10.1186/s12951-026-04767-7

**Published:** 2026-07-03

**Authors:** Kai Yang, Pei Zou, Yikun Ju, Shuai Zhu, Hongli Zhao, Jiandong Ye, Naihsin Hsiung, Ruilin Liu, Qingchun Song, Xiancheng Wang

**Affiliations:** 1 Department of Plastic and Aesthetic (Burn) Surgery, The Second Xiangya Hospital, Central South University, Changsha, 410011 China; 2 College of Materials Science and Engineering, Hunan University, Changsha, 410082 China; 3 Department of Cardiovascular Surgery, The Second Xiangya Hospital of Central South University, Central South University, Changsha, China

**Keywords:** Pyroptosis, Perforator flap, Adhesive hydrogel, Exosomes

## Abstract

Multiterritory perforator flaps are widely used in reconstructive surgery; however, distal regions, particularly the choke zone, remain highly susceptible to necrosis due to sustained ischemia, oxidative stress, and endothelial inflammatory injury that impair microcirculatory remodeling. Although stem cell-derived exosomes are promising cell-free therapeutics, their efficacy in ischemic flaps is often limited by poor local retention and rapid loss of bioactivity. Here, we develop a bioadhesive antioxidant hydrogel composed of oxidized hyaluronic acid-dopamine and sericin methacrylate (SerMA) to enable stable wet-tissue adhesion and sustained local delivery of hypoxia-preconditioned adipose-derived stem cell exosomes. This strategy attenuates oxidative stress, suppresses NLRP3 inflammasome activation and endothelial pyroptosis, and preserves mitochondrial and angiogenic function under ischemic conditions. In a rat multiterritory perforator flap model, local application of the exosome-loaded hydrogel significantly enhanced flap perfusion and survival, promoted vascular remodeling within the choke zone, and reduced inflammatory responses. Transcriptomic analysis further revealed suppression of inflammasome-associated pathways and enrichment of vascular remodeling programs. Collectively, this work presents a bioadhesive hydrogel–based exosome delivery strategy that stabilizes the ischemic microenvironment and improves outcomes in complex perforator flap reconstruction.

**Graphical Abstract:**

**Figure:**
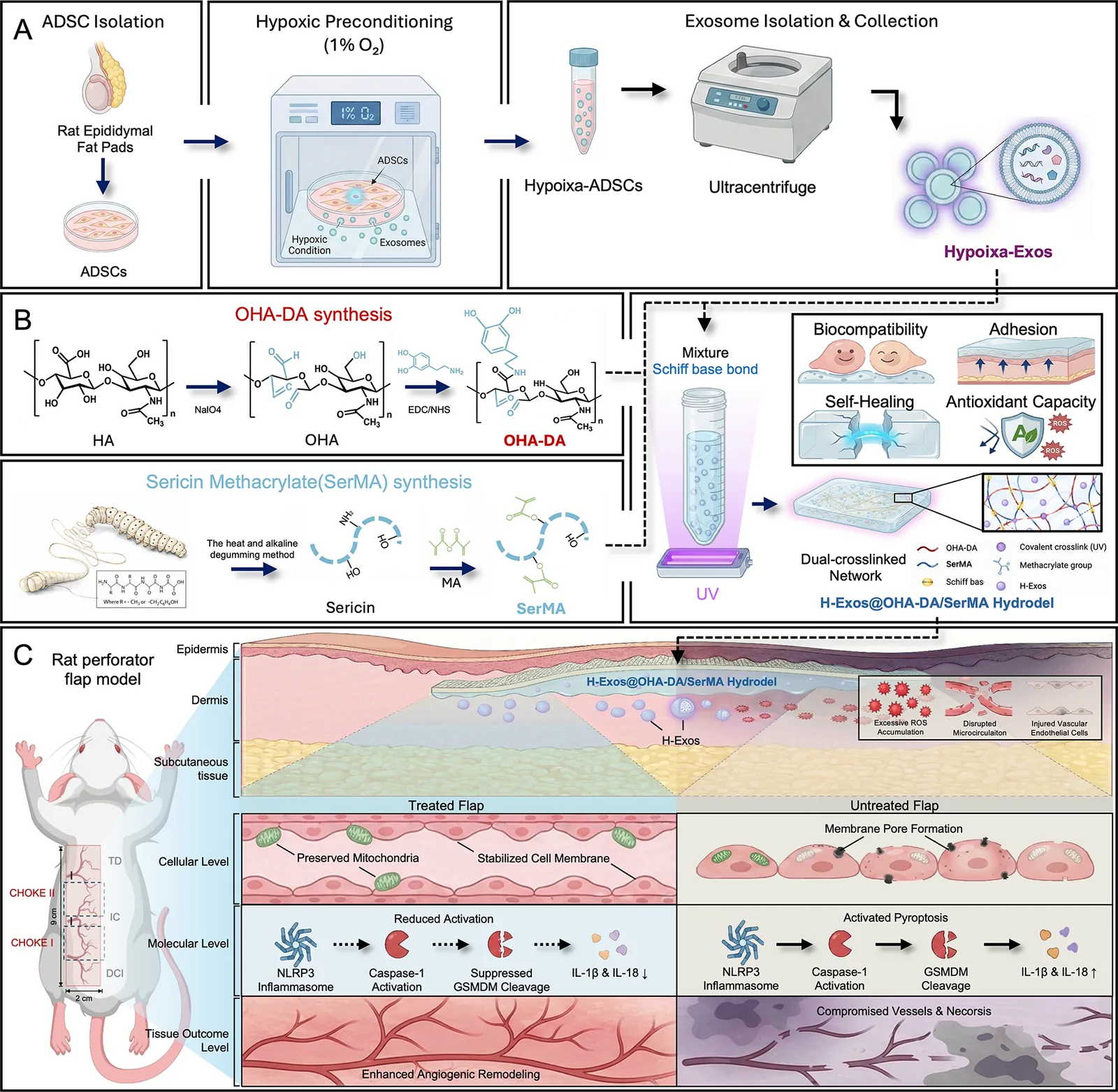

**Supplementary Information:**

The online version contains supplementary material available at https://doi.org/10.1186/s12951-026-04767-7.

## Background

Perforator flaps are commonly used in reconstructive and microsurgical procedures because of their superior tissue matching and reduced donor-site morbidity [1–3]. However, distal flap necrosis remains a persistent and clinically challenging complication, particularly in multiterritory perforator flaps, where perfusion across adjacent vascular territories depends on choke vessels with limited adaptive capacity [4–7]. Insufficient blood flow in these regions causes sustained ischemia, excessive oxidative stress, and progressive microcirculatory dysfunction, ultimately compromising flap viability and functional outcomes [7–9].

Endothelial pyroptosis is a pivotal mechanism underlying ischemic flap failure, particularly in impaired microcirculatory adaptation. Unlike apoptosis, pyroptosis is a highly inflammatory form of programmed cell death characterized by NLRP3 inflammasome activation and caspase-1-dependent gasdermin D (GSDMD) cleavage, leading to endothelial barrier disruption, vascular leakage, and loss of microvascular integrity under ischemic conditions [10–18]. Excessive oxidative stress is a critical upstream trigger of endothelial pyroptosis. Persistent ischemia-induced overproduction of reactive oxygen species (ROS) disrupts endothelial homeostasis, amplifies inflammatory signaling, and promotes inflammasome activation, thereby coupling oxidative injury to pyroptotic endothelial dysfunction and impaired angiogenic remodeling [19–21]. Despite the increasing confirmation of endothelial pyroptosis in ischemic vascular pathology, therapeutic strategies that directly target this process within the hostile multiterritory perforator flap microenvironment remain limited [22, 23].

Mesenchymal stem cell-derived exosomes (Exos) have attracted attention as cell-free therapeutics for ischemic tissue repair due to their abilities to modulate inflammation, promote angiogenesis, and enhance cell survival through paracrine signaling [24–28]. Among them, hypoxia-preconditioned adipose-derived stem cell (ADSC) exosomes (H-Exos) exhibit superior bioactivity compared with that of normoxic exosomes (N-Exos), largely due to the enrichment of hypoxia-responsive cargos. Particularly, hypoxic preconditioning is associated with enhanced hypoxia-inducible factor-1α (HIF-1α)-related signaling, a central regulator of endothelial adaptation to hypoxia that links redox homeostasis, mitochondrial function, and inflammatory cell death pathways. In addition to cargo composition, H-Exos are more efficiently internalized by endothelial cells than N-Exos, enabling more effective delivery of therapeutic signals to the vascular endothelium, a key cellular target for microcirculatory remodeling and flap survival [29–32]. These features suggest that H-Exos are intrinsically well suited for modulating endothelial fate and suppressing oxidative stress-associated pyroptosis within ischemic tissues. However, despite these advantages, the clinical translation of exosome-based therapies remains limited by rapid clearance, insufficient tissue retention, and bioactivity loss following direct administration into hostile ischemic microenvironments [33–36].

Hydrogel-based delivery systems offer a promising strategy to overcome these limitations by enabling localized retention and sustained release of bioactive cargos [37–40]. Compared with direct injection or systemic administration, hydrogels provide a confined therapeutic niche that improves exosome stability and bioavailability, particularly in ischemic and inflammatory tissues. Among them, catechol-functionalized hydrogels have attracted particular interest due to their strong wet-tissue adhesion and intrinsic antioxidant properties, stably anchoring to moist tissue surfaces and scavenging excessive ROS, respectively [41–43]. These features are especially advantageous for ischemic flap applications, where tissue motion, interstitial fluid exchange, and oxidative stress collectively undermine local therapeutic efficacy [44, 45]. However, the development of adhesive antioxidant hydrogels that are simultaneously injectable, mechanically stable, and capable of controlled exosome release remains challenging, and their potential to modulate endothelial injury pathways in complex perforator flap settings has not been fully explored.

Native hyaluronic acid and sericin possess favorable biocompatibility and biodegradability for biomedical applications; however, their intrinsic physicochemical properties are insufficient to simultaneously achieve wet-tissue adhesion, injectable gelation, mechanical stability, and sustained local retention and diffusion-regulated release behavior. Therefore, functional modification strategies were introduced to endow these biomaterials with additional bioactive and structural properties. Specifically, oxidized hyaluronic acid conjugated with dopamine (OHA-DA) provides catechol-mediated wet adhesion, antioxidant activity, and dynamic Schiff-base interactions, whereas sericin methacrylate (SerMA), generated through methacrylation of sericin, introduces photocrosslinkable methacrylate groups that enable rapid in situ covalent network formation and mechanical stabilization [46]. Accordingly, the present hydrogel system was designed based on the functional integration of chemically modified OHA-DA and SerMA rather than simple blending of native HA and sericin. Importantly, through compositional optimization (OHA-DA/SerMA = 1:0.2), this dual-network system achieves a balanced combination of adhesion, antioxidant capacity, injectability, self-healing behavior, and mechanical stability, enabling effective stabilization of the hostile ischemic microenvironment. Mechanistically, dynamic Schiff-base interactions between aldehyde groups of OHA-DA and amino groups derived from SerMA enable rapid initial gelation and reversible network rearrangement, contributing to injectability and self-healing behavior. Meanwhile, catechol groups introduced through dopamine conjugation provide strong wet-tissue adhesion and antioxidant activity, thereby enhancing local tissue retention under ischemic conditions. In parallel, photocrosslinkable methacrylate groups within SerMA form a stable covalent network upon light activation, reinforcing structural integrity and reducing premature exosome diffusion. Through the cooperative effects of dynamic and covalent dual-network crosslinking, the dual-network architecture enables sustained exosome retention and controlled release kinetics that are difficult to achieve through simple physical blending or conventional injection-based delivery systems. However, whether this adhesive antioxidant hydrogel can serve as an effective delivery platform for H-Exos and simultaneously modulate endothelial pyroptosis in multiterritory perforator flaps is unknown. Therefore, characterization of both in vitro release behavior and in vivo retention kinetics is essential for validating the hydrogel as a sustained exosome delivery platform.

**Fig. 1:**
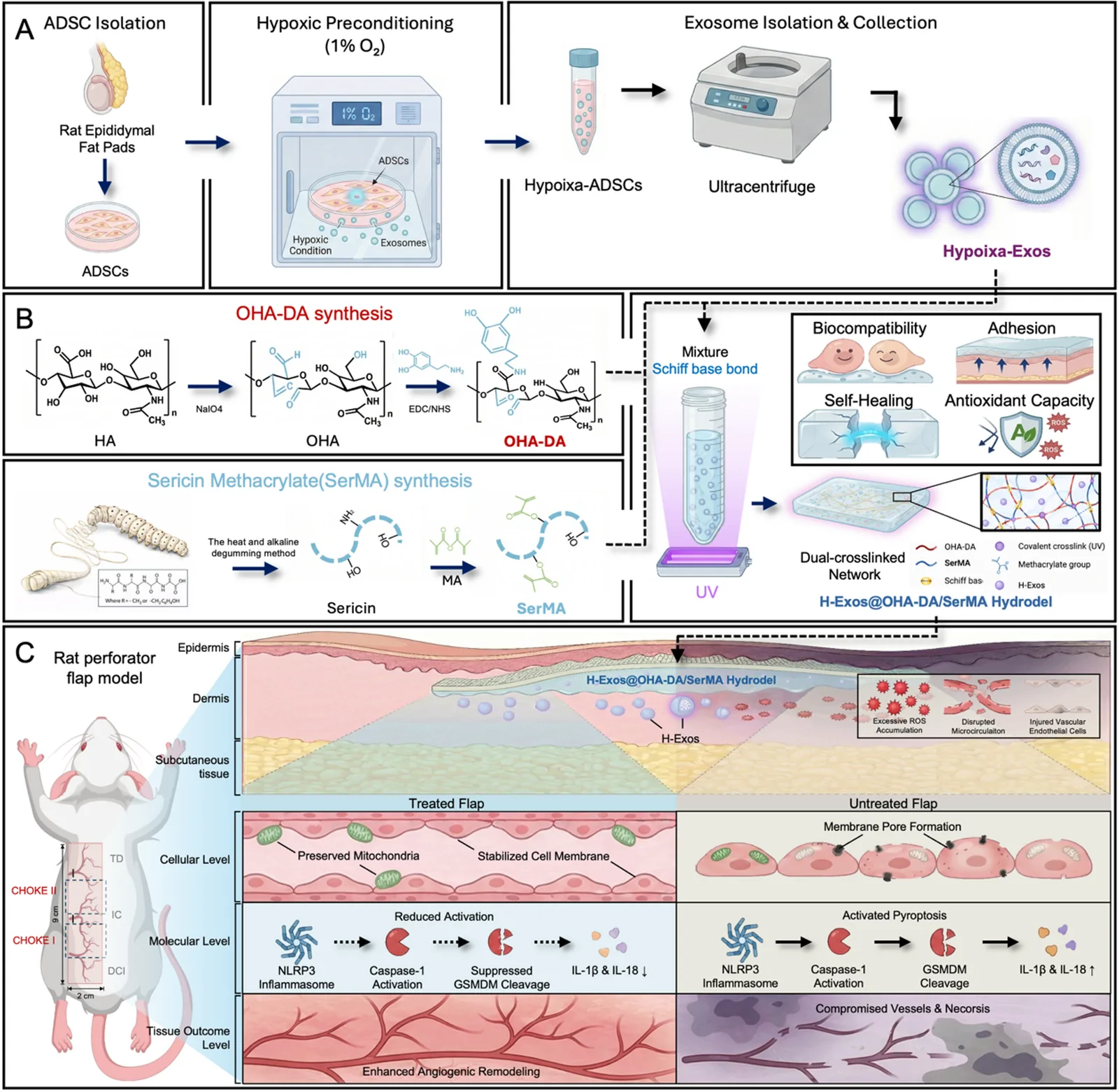
Design and therapeutic mechanism of H-Exos@OHA-DA/SerMA hydrogel for multiterritory perforator flaps. (**A**) ADSCs were isolated from rat epididymal fat pads and cultured under hypoxic conditions (1% O₂) to obtain hypoxia-preconditioned exosomes (H-Exos), which were collected by ultracentrifugation. (**B**) OHA-DA was synthesized by dopamine conjugation to oxidized hyaluronic acid, and SerMA was obtained by methacrylation of sericin extracted from silkworm cocoons. H-Exos were encapsulated within the OHA-DA/SerMA hydrogel formed through dynamic Schiff-base bonding and UV-initiated photo-crosslinking. Yielding an injectable, self-healing hydrogel with tissue adhesion, antioxidant activity, and sustained exosome release. (**C**) After topical application to the choke zone of a rat multiterritory perforator flap, H-Exos@OHA-DA/SerMA hydrogel scavenges excessive ROS, enhances local exosome retention, preserves mitochondrial integrity, and stabilizes endothelial cell membranes. At the molecular level, the hydrogel suppresses NLRP3 inflammasome activation, Caspase-1 activation, and GSDMD-mediated pyroptosis, leading to reduced IL-1β and IL-18 release, enhanced angiogenic remodeling, and improved flap perfusion and survival. All schematic illustrations were created by the authors and are original, with no reuse or modification of previously published copyrighted material

In this study, we engineered a multifunctional double-crosslinked hydrogel integrating OHA-DA and SerMA and its effects were examined in a ischemia-induced rat multiterritory perforator flap and oxidative injury human umbilical vein endothelial cell (HUVEC) model. The microenvironment-oriented exosome delivery strategy integrates hypoxia-conditioned exosomes with a bioadhesive antioxidant hydrogel **(**Fig. 1**).** H-Exos were selected as the therapeutic payload, based on their enhanced adaptability to ischemic stress and capacity to modulate endothelial injury pathways [33, 47]. Rather than representing a simple material combination, the dual-functionalized OHA-DA/SerMA network was engineered as an active microenvironment-regulating matrix, enabling stable wet-tissue adhesion, redox modulation, injectability, and sustained local exosome retention through a dual-crosslinked network. This system was locally applied to the choke zone of a rat multiterritory perforator flap model, characterized by severe ischemia, oxidative stress, and endothelial vulnerability [15, 48]. Distinct from conventional exosome delivery or standalone antioxidant materials, this design conceptually links localized bioadhesion, microenvironment stabilization, and targeted modulation of ischemic endothelial injury within a single cell-free platform. Through this integrated framework, the present study investigates whether controlling the ischemic microenvironment can confirm the therapeutic potential of hypoxia-conditioned exosomes and improve microvascular adaptation in complex perforator flap reconstruction.

## Methods

### Materials

HA (Mw ≈ 1000 kDa) was purchased from Sigma-Aldrich (St. Louis, MO, USA). Dopamine hydrochloride, sodium periodate (NaIO₄), methacrylic anhydride, and lithium phenyl-2,4,6-trimethylbenzoylphosphinate (LAP) were obtained from Sigma-Aldrich and used as is unless otherwise stated. Silkworm cocoons (*Bombyx mori*) were purchased from Jiangsu Province (China). HUVECs were obtained from the Cell Bank of the Chinese Academy of Sciences (Shanghai, China). Male Sprague–Dawley rats (8–10 weeks old, 250–300 g) were purchased from Hunan SJA Laboratory Animal Co. Ltd. (Changsha, China). ADSCs were isolated from epididymal adipose tissue of male Sprague–Dawley rats. All animal experiments were approved by the Ethics Committee for Animal Experimentation of the Second Xiangya Hospital of Central South University (approval no. 20240567) and performed in accordance with the ARRIVE guidelines. Cell culture media, fetal bovine serum, penicillin-streptomycin, phosphate-buffered saline (PBS), and other routine reagents were purchased from Thermo Fisher Scientific (Waltham, MA, USA) unless otherwise specified.

### Preparation of OHA-DA

Oxidized hyaluronic acid (OHA) was synthesized according to a previously reported method with minor modifications. Briefly, hyaluronic acid (HA, 1.0 g) was dissolved in 100 mL of deionized water and stirred overnight until completely dissolved. Sodium periodate (NaIO₄, 0.54 g, corresponding to a molar ratio of 20% relative to the HA repeating units) was then added, and the reaction was allowed to proceed in the dark at room temperature (25 °C) for 6 h. Subsequently, 1 mL of ethylene glycol was added to quench excess NaIO₄, and the reaction mixture was stirred for an additional 1 h. The resulting solution was dialyzed against deionized water (MWCO 3500 Da) for 3 days and lyophilized to obtain OHA.

For dopamine conjugation, OHA (500 mg) was dissolved in 50 mL of MES buffer (0.1 M, pH 5.5). EDC (192 mg) and NHS (115 mg) were added and allowed to react for 30 min to activate the carboxyl groups. Dopamine hydrochloride (95 mg) was subsequently added, and the reaction was carried out under gentle stirring for 24 h in the dark at room temperature. The resulting OHA-DA conjugate was purified by dialysis against deionized water (MWCO 3500 Da) for 3 days and then lyophilized for further use.

The successful oxidation of HA and dopamine conjugation were confirmed by ^1H NMR and FT-IR analyses.

### Preparation of SerMA

Sericin methacrylate (SerMA) was synthesized through methacrylation of sericin extracted from natural silkworm cocoons, as previously reported with minor modifications [49, 50]. Briefly, natural silkworm cocoons (10 g) were washed with deionized water and boiled in 400 mL of 0.02 M Na₂CO₃ solution for 1 h to remove insoluble silk fibroin. The resulting sericin solution was collected after centrifugation (1500 × g, 10 min) and filtration to remove residual impurities. Subsequently, the sericin solution was dialyzed against deionized water using dialysis tubing (MWCO 3.5 kDa) for 4 days to remove Na₂CO₃ and other low-molecular-weight impurities, followed by lyophilization to obtain purified sericin.

For methacrylation, purified sericin (5%, w/v) was dissolved in deionized water and maintained at 50 °C under continuous stirring. Methacrylic anhydride was slowly added dropwise at a volume ratio of 0.5 mL per gram of sericin and allowed to react for 6 h. The reaction mixture was subsequently dialyzed against deionized water (MWCO 3.5 kDa) for 4 days to remove residual methacrylic anhydride and reaction by-products. The purified solution was then freeze-dried to obtain SerMA. Successful methacrylation was confirmed by ^1H NMR and FT-IR analyses.

### Crosslinking mechanism of OHA-DA/SerMA hydrogel

The OHA-DA/SerMA hydrogel was constructed through a dual-crosslinking strategy consisting of dynamic Schiff-base bonding and photo-induced polymerization. Specifically, aldehyde groups on oxidized hyaluronic acid-dopamine (OHA-DA) reacted with amino groups present in sericin methacrylate (SerMA), forming reversible Schiff-base linkages that contributed to rapid gel formation, injectability, and self-healing behavior. Subsequently, methacrylate groups on SerMA underwent free-radical polymerization under 405 nm light irradiation in the presence of the LAP photoinitiator, generating a stable covalently crosslinked network that enhanced the mechanical strength and structural stability of the hydrogel. UV irradiation was applied using a 405 nm curing lamp (intensity: 30 mW/cm²) positioned 10 cm above the sample surface for 60 s.

In addition, catechol groups introduced by dopamine conjugation provided strong wet-tissue adhesion through hydrogen bonding, π–π interactions, and covalent interactions with tissue surfaces. These catechol moieties also endowed the hydrogel with intrinsic antioxidant activity through reactive oxygen species scavenging. The combination of dynamic and permanent crosslinking networks enabled the hydrogel to simultaneously achieve injectability, self-healing capability, mechanical robustness, tissue adhesion, and sustained exosome retention.

### Preparation of OHA-DA/SerMA hydrogel and H-Exos@Hydrogel

OHA-DA and SerMA were dissolved in PBS at predetermined concentrations and mixed thoroughly with the LAP photoinitiator. H-Exos were added to the precursor solution and gently mixed prior to gelation to ensure homogeneous distribution. The resulting mixture was exposed to blue light (405 nm) for 60 s to induce photo-crosslinking, forming the OHA-DA/SerMA hydrogel in situ. Hydrogels without exosomes (Gel) or loaded with N-Exos (Gel@N-Exos) were simultaneously prepared as controls.

### In vitro exosome release assay

To evaluate the sustained release behavior of exosomes from the hydrogel, H-Exos-loaded OHA-DA/SerMA hydrogels were incubated in PBS at 37 °C under gentle shaking. At predetermined time points (6 h, 12 h, 1 d, 3 d, 5 d, 7 d, 10 d, and 14 d), the supernatants were collected and replaced with an equal volume of fresh PBS. The amount of released exosomes was quantified using a bicinchoninic acid (BCA) protein assay. Cumulative release profiles were calculated based on the total released exosomal protein content over time.

### In vivo exosome retention and fluorescence imaging

To investigate local retention of exosomes in vivo, H-Exos were labeled using a DiR fluorescent dye according to the manufacturer’s protocol. DiR-labeled H-Exos (DiR-H-Exos) or hydrogel-loaded DiR-H-Exos (Gel@DiR-H-Exos) were applied to the choke zone of multiterritory perforator flaps immediately after flap elevation. Fluorescence signals were monitored using an in vivo imaging system (IVIS) at postoperative Days 0, 1, 2, 3, 4, 5, 6, and 7. Quantitative analysis of fluorescence intensity within the flap region was performed using Living Image software.

### Hydrogel characterization

The internal morphology of the hydrogels was characterized by scanning electron microscopy (SEM). Prior to SEM observation, hydrogel samples were rapidly frozen in liquid nitrogen and subsequently lyophilized to minimize structural collapse and dehydration-induced artifacts associated with ice crystal formation. The freeze-dried hydrogels were fractured, sputter-coated with gold, and imaged using SEM. Quantitative analysis of pore size and porosity was performed based on SEM images. It should be noted that the observed porous morphology primarily represents the microstructure of the lyophilized hydrogels rather than the fully hydrated native state. Therefore, SEM analysis was mainly used for comparative evaluation of relative network morphology among different hydrogel formulations.

^1H NMR spectroscopy and Fourier transform infrared (FT-IR) spectroscopy were performed to characterize the chemical structures of OHA-DA, SerMA, and the double-crosslinked hydrogel system. Peak assignments were interpreted according to previously reported dopamine-functionalized hyaluronic acid derivatives, methacrylated sericin biomaterials, and OHA-based Schiff-base hydrogels [49–51].

Rheological measurements were performed using a rotational rheometer equipped with a 20 mm parallel-plate geometry at a gap distance of 1.0 mm. Hydrogel samples were first photocrosslinked under 405 nm light irradiation for 60 s and subsequently loaded onto the rheometer plate for characterization. All rheological measurements were performed at 37 °C. To minimize solvent evaporation, the exposed sample edge was sealed with low-viscosity silicone oil throughout the measurements. Time-sweep measurements were conducted at a constant strain of 1% and frequency of 1 Hz to evaluate gelation kinetics during photo-crosslinking. Shear-thinning behavior was evaluated over a shear-rate range of 0.1–100 s⁻¹ using a continuous unidirectional sweep. Each shear rate was maintained for 10 s before data acquisition. Strain-sweep measurements were performed over a strain range of 0.1–10% at a fixed frequency of 1 Hz. Frequency-sweep measurements were conducted over an angular frequency range of 0.1–70 rad/s at a constant strain of 1%, which was within the linear viscoelastic region. Self-healing behavior was evaluated using cyclic step-strain measurements by alternately applying low strain (1%) and high strain (400%) at a constant frequency of 1 Hz. Each strain step was maintained for 60 s. All rheological measurements were performed in triplicate.

The injectability and self-healing behavior of the hydrogels were visually evaluated by extrusion through a syringe and macroscopic reconstruction after fragmentation.

Swelling behavior was assessed by incubating hydrogels in PBS at 37 °C and recording mass changes over time. Mechanical properties were evaluated using uniaxial compression testing. Wet-tissue adhesive strength was assessed using lap-shear testing on biological substrates.

The antioxidant capacity of the hydrogels was evaluated through hydroxyl radical and superoxide anion scavenging assays. In vitro degradation behavior was evaluated by incubating hydrogels in PBS at 37 °C and monitoring mass loss over time.

### Isolation and characterization of H-Exos

ADSCs were cultured under normoxic or hypoxic conditions (1% O₂) for 24 h as previously described [^52^]. Conditioned media were collected and sequentially subjected to differential centrifugation (300 × g for 10 min, 2,000 × g for 10 min, and 10,000 × g for 30 min) at 4 °C to remove cells and debris. The resulting supernatant was filtered through a 0.22-µm membrane to eliminate residual large vesicles and particles. Exosomes were then isolated using ultracentrifugation at 100,000 × g for 70 min at 4 °C. The exosome pellet was resuspended in sterile PBS and washed using a second ultracentrifugation step at 100,000 × g for 70 min at 4 °C. The final exosome pellet was resuspended in PBS and stored at − 80 °C for subsequent experiments. The morphology of isolated exosomes was examined using transmission electron microscopy (TEM), and particle size distribution was analyzed using nanoparticle tracking analysis (NTA). Exosomal identity was further verified by western blotting of canonical exosomal markers, including CD63, HSP70, and TSG101. To evaluate the effect of hypoxic preconditioning, the expression of HIF-1α in exosomes was assessed at both the protein and mRNA levels using western blotting and quantitative real-time PCR, respectively, following RNA extraction. For all experiments, exosome dosage was normalized to the total protein content measured using a bicinchoninic acid (BCA) assay.

### In vitro cell experiments

HUVECs were cultured under standard normoxic conditions. Oxidative stress injury was induced by treatment with H₂O₂ for the indicated durations to establish an in vitro oxidative injury model. Cell viability was assessed using the CCK-8 assay and cell proliferation was evaluated using EdU incorporation. Intracellular ROS levels were detected using the DCFH-DA probe and mitochondrial ROS generation was assessed using MitoSOX staining. Mitochondrial membrane potential (ΔΨm) was evaluated using JC-1 staining. Endothelial migratory capacity and angiogenic behavior were assessed using scratch wound healing and Matrigel tube formation assays, respectively. Ultrastructural alterations of endothelial cells, including mitochondrial damage and plasma membrane pore formation, were examined using TEM. Pyroptosis-related proteins, including NLRP3, caspase-1, and GSDMD, were analyzed using IF staining and western blotting, and the secretion of inflammatory cytokines was quantified using ELISA.

### Western blotting

Cells or tissue samples were lysed using RIPA lysis buffer supplemented with protease inhibitors and phenylmethylsulfonyl fluoride. Protein concentrations were determined using a BCA protein assay. Equal amounts of protein were separated using 12% SDS-polyacrylamide gels and transferred onto polyvinylidene difluoride membranes. Membranes were blocked with 5% non-fat milk in PBST at 25 °C for 3 h and incubated with primary antibodies at 4 °C overnight. The following primary antibodies were used: anti-HIF-1α (cat. no. ab308433, 1:1000; Abcam, Cambridge, UK), anti-Caspase-1 (cat. no. 83383, 1:1000; Cell Signaling Technology, Danvers, MA, USA), anti-GSDMD (cat. no. ab219800, 1:2000; Abcam), anti-NLRP3 (cat. no. ab314905, 1:1000; Abcam), anti-ASC/TMS1 (cat. no. 67824, 1:1000; Cell Signaling Technology), and anti-GAPDH (cat. no. 60004-1-Ig, 1:50000; Proteintech, Wuhan, China). After washing, membranes were incubated with HRP-conjugated goat anti-rabbit IgG (cat. no. SA00001-2, 1:6000; Proteintech) or goat anti-mouse IgG (cat. no. SA00001-1, 1:6000; Proteintech) at 25 °C for 1 h. Protein bands were visualized using SuperSignal™ West Pico PLUS chemiluminescent substrate (cat. no. 34577; Thermo Fisher Scientific) and imaged with a ChemiScope 6100 chemiluminescence imaging system (Qinxiang Scientific Instrument Co., Shanghai, China). Band intensities were quantified using ImageJ software (Version: 2.14.0/1.54f) and normalized to GAPDH.

### Immunofluorescence staining

Cells cultured on coverslips were fixed with 4% paraformaldehyde (cat. no. P1110; Solarbio, Beijing, China) at 25 °C for 15 min, permeabilized with 0.3% Triton X-100 (cat. no. P1080; Solarbio) for 30 min, and blocked with 5% bovine serum albumin at 37 °C for 1 h. Cells were then incubated with primary antibodies at 4 °C overnight. For detection of pyroptosis-related proteins, anti-GSDMD antibody (cat. no. 20770-1-AP, 1:250; Proteintech) was used. After washing with PBS, cells were incubated with CoraLite594-conjugated goat anti-rabbit IgG (H + L) secondary antibody (cat. no. SA00013-4, 1:100; Proteintech) at 37 °C for 90 min in the dark. Nuclei were counterstained with DAPI (cat. no. C1002; Beyotime Biotechnology, Shanghai, China) for 10 min. Fluorescence images were acquired using a fluorescence microscope (MF-53; Mingmei Technology Co., Guangzhou, China). Quantitative analysis of fluorescence intensity was performed using ImageJ software.

### *ELISA*

The concentrations of inflammatory cytokines were quantified using ELISA kits according to the manufacturers’ instructions. Human interleukin-1β (IL-1β) levels were measured using the Human IL-1 beta Uncoated ELISA Kit (cat. no. 88-7261; Invitrogen, Thermo Fisher Scientific). Human total interleukin-18 (IL-18) levels were determined using the Human Total IL-18 Sandwich ELISA Kit (cat. no. KE00193; Proteintech). Absorbance was measured using a microplate reader, and cytokine concentrations were calculated based on standard curves generated for each assay.

### Animal model of multiterritory perforator flap

A multiterritory perforator flap was established on the right dorsal region of male Sprague–Dawley rats. Rats were anesthetized using isoflurane inhalation (induction: 4–5%; maintenance: 1.5–2.0%) delivered with oxygen. The medial border of the flap was defined by the posterior midline, inferior border by the posterior superior iliac spine, superior border by the subscapular angle, and lateral border parallel to the medial border. The flap encompassed three perforator territories supplied by the thoracodorsal (TD), posterior intercostal (IC), and deep circumflex iliac (DCI) arteries, including the intervening choke I and choke II zones. The flap size was standardized to 9.0 × 2.0 cm. After incision of the skin and subcutaneous tissue down to the superficial layer of the deep fascia, the subdermal vascular plexus was carefully preserved. The TD and posterior IC artery perforators were identified and ligated, while the DCI artery perforator was preserved as the sole vascular supply, thereby establishing a multiterritory perforator flap model with well-defined choke zones. Following complete elevation of the flap and confirmation of ischemia in the choke zone region, 1.0 mL of hydrogel precursor solution (Gel, Gel@N-Exo, or Gel@H-Exo) was evenly applied to the flap bed, with particular emphasis on the choke II zone. The flap was then repositioned and exposed to visible light for 60 s to induce in situ photocrosslinking, followed by closure with interrupted 4 − 0 non-absorbable sutures. Postoperatively, rats were housed individually and monitored daily [48].

### Histological and immunohistochemical (IHC) analysis

Flap tissues harvested from the choke zone were fixed in paraformaldehyde, embedded in paraffin, and sectioned for histological analysis. Hematoxylin and eosin (H&E) and Masson’s trichrome staining were performed to evaluate tissue morphology, inflammatory infiltration, and collagen deposition. IHC staining was conducted to assess angiogenesis- and inflammation-related markers, including CD34, VEGF, α-SMA, and IL-1β. IF staining was performed to detect pyroptosis-related proteins, including caspase-1 and GSDMD. For quantitative analysis of IHC and IF staining, choke-zone tissues were collected from the central region of the choke II zone. A total of five biological replicates were included in each group. For each sample, three non-consecutive paraffin sections were selected, and three to five randomly chosen high-power fields were captured from each section under identical microscope settings. Image acquisition parameters, including exposure time, magnification, and threshold settings, were kept consistent across all groups. Quantification was performed using ImageJ software by an investigator blinded to group allocation. For IHC staining, CD34-positive microvessel density and the positive staining areas of VEGF, α-SMA, and IL-1β were quantified. For IF staining, the mean fluorescence intensity and the proportion of cleaved Caspase-1– or N-GSDMD–positive signals within CD31-positive vascular regions were quantified. In addition, endothelial proliferation was evaluated by Ki67/CD31 double immunofluorescence staining, and the percentage of Ki67⁺/CD31⁺ cells relative to total CD31⁺ endothelial cells was calculated.

### Transcriptomic analysis

Total RNA was extracted from choke-zone flap tissues using TRIzol reagent (Invitrogen, Carlsbad, CA, USA) according to the manufacturer’s protocol. RNA integrity and quality were assessed prior to sequencing. High-throughput RNA sequencing (RNA-seq) was performed on an Illumina platform. differentially expressed genes (DEGs) were identified based on fold change and adjusted p-values following data normalization and statistical analysis. Gene Ontology (GO) biological process enrichment and Kyoto Encyclopedia of Genes and Genomes (KEGG) pathway analyses were performed using standard bioinformatics pipelines to interpret the functional significance of DEGs.

### Statistical analysis

All quantitative data are presented as mean ± standard deviation. Statistical analyses were performed using GraphPad Prism software. Comparisons among multiple groups were conducted using one-way analysis of variance followed by appropriate post-hoc tests. Data distribution was assumed to be normal unless otherwise stated. A p-value < 0.05 was considered statistically significant.

## Result

### Fabrication and physicochemical characterization of OHA-DA/SerMA hydrogel

**Fig. 2:**
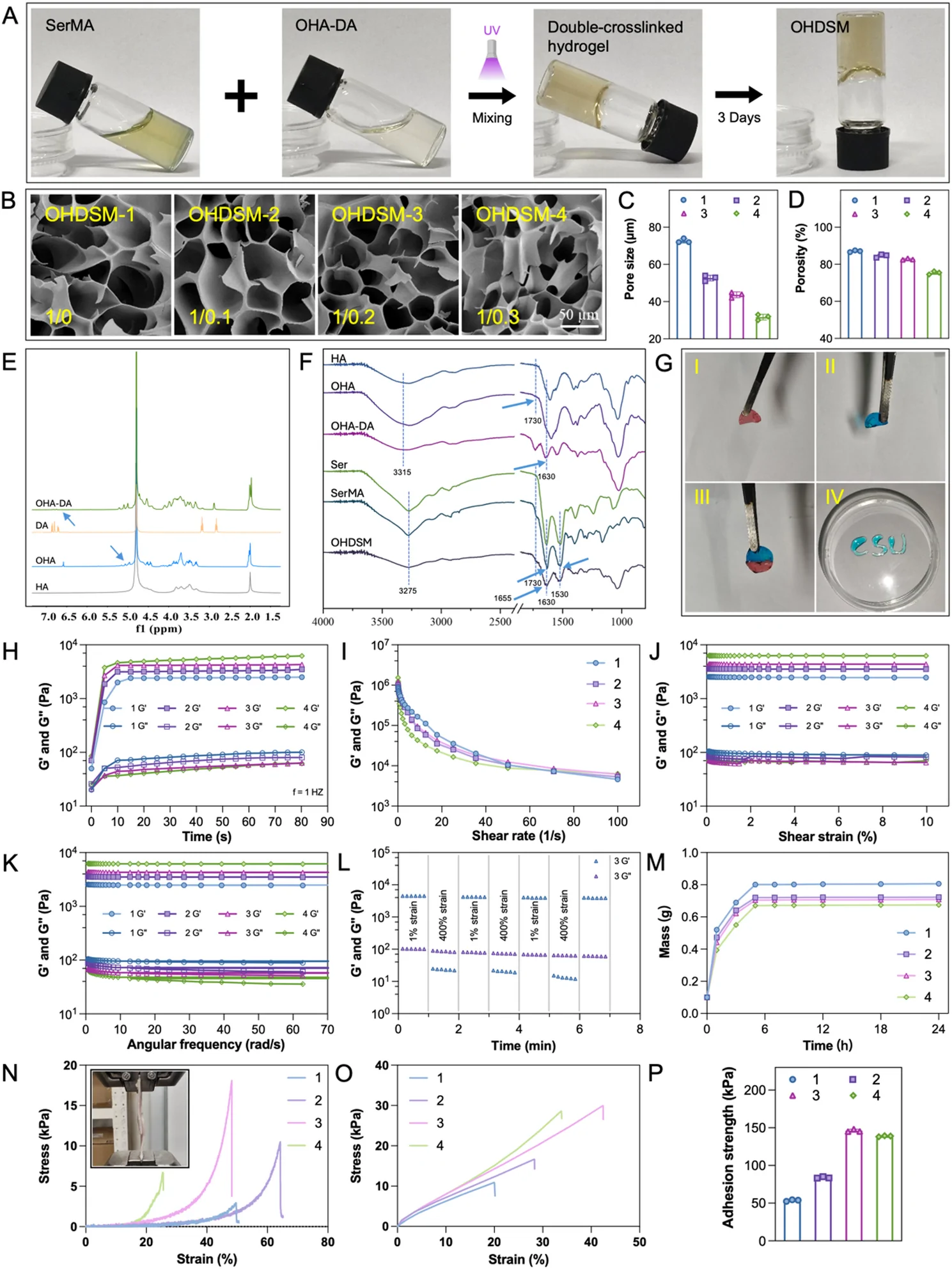
Fabrication and physicochemical characterization of the OHA-DA/SerMA hydrogel. (**A**) Representative photographs showing SerMA and OHA-DA precursor solutions, hydrogel formation following mixing and 405 nm light irradiation, and structural integrity of the hydrogel after incubation for 3 days. (**B**) Scanning electron microscopy (SEM) images of lyophilized OHA-DA/SerMA hydrogels with different compositions, denoted as groups 1–4 (OHDSM-1 to OHDSM-4), corresponding to OHA-DA/SerMA mass ratios of 1:0, 1:0.1, 1:0.2, and 1:0.3, respectively, revealing relatively interconnected porous morphologies after freeze-drying. Scale bar: 50 μm. (**C**–**D**) Quantitative analysis of pore size (**C**) and porosity (**D**) of hydrogels with different compositions. (**E**) 1 H NMR spectra of HA, OHA, DA, OHA-DA, Ser, and SerMA. The appearance of hemiacetal proton signals at 4.9–5.2 ppm confirmed HA oxidation, while characteristic dopamine aromatic (6.7 ppm) and methylene (2.88 ppm) proton signals verified successful dopamine conjugation. Newly emerged vinyl proton peaks at 5.7 and 6.1 ppm and methyl proton signals at 1.9 ppm confirmed successful methacrylation of sericin. (**F**) FT-IR spectra of HA, OHA, OHA-DA, sericin (Ser), sericin methacrylate (SerMA), and the OHA-DA/SerMA hydrogel, confirming successful chemical modification and the presence of characteristic functional groups associated with methacrylate modification and Schiff-base interactions. (**G**) Representative photographs demonstrating injectability, self-healing behavior, and shape adaptability of the hydrogel. (**H**) Time-sweep rheological analysis performed at 1% strain and 1 Hz frequency, showing rapid gelation behavior following 405 nm light irradiation, with storage modulus (G′) rapidly exceeding loss modulus (G″). (**I**) Shear-thinning behavior measured over a shear-rate range of 0.1–100 s⁻¹. (**J**) Strain-dependent viscoelastic behavior measured at a fixed frequency of 1 Hz. (**K**) Frequency-dependent rheological behavior measured at a constant strain of 1% within the linear viscoelastic region. (**L**) Cyclic step-strain measurements alternating between 1% and 400% strain, demonstrating rapid recovery of viscoelastic properties and self-healing capability. (**M**) Swelling behavior of hydrogels with different compositions over time. (**N**) Representative stress–strain curves obtained from wet-tissue lap-shear adhesion testing, with the experimental setup shown in the inset. (**O**) Compressive stress–strain curves of OHA-DA/SerMA hydrogels with different compositions. (**P**) Quantification of wet-tissue adhesion strength, indicating that OHDSM-3 (OHA-DA/SerMA = 1:0.2) exhibited the optimal balance between mechanical robustness and interfacial adhesion

The OHA-DA/SerMA hydrogel was fabricated through a dual-crosslinking strategy involving dynamic Schiff-base bonding between aldehyde groups of OHA-DA and amino groups of SerMA, together with UV-triggered free-radical polymerization of methacrylate groups on SerMA. This dual-network architecture combines the injectability and self-healing characteristics of dynamic covalent bonds with the mechanical stability provided by permanent photo-crosslinked networks. As shown in Fig. 2A, liquid precursor solutions of SerMA and OHA-DA were homogeneously mixed and rapidly transformed into a free-standing hydrogel upon UV irradiation. The inverted-vial test demonstrated successful gel formation and self-supporting behavior, indicating efficient in situ gelation [49].

To evaluate the effect of composition, four hydrogel formulations with increasing OHA-DA content were prepared and designated as OHDSM-1, OHDSM-2, OHDSM-3, and OHDSM-4, corresponding to OHA-DA/SerMA mass ratios of 1:0, 1:0.1, 1:0.2, and 1:0.3, respectively (Fig. 2B).

Macroscopic handling properties of the hydrogels were examined. As shown in Fig. 2G (I–III), two separately dyed hydrogel blocks rapidly rejoined into a continuous structure after contact, demonstrating self-healing behavior. In addition, the hydrogel could be smoothly extruded through a syringe and retained its integrity after injection (Fig. 2G (IV)), confirming its injectability.

The microstructure of the lyophilized hydrogels was analyzed by scanning electron microscopy (SEM). All formulations exhibited relatively interconnected porous morphologies after freeze-drying (Fig. 2B). Although partial structural alteration caused by dehydration during lyophilization cannot be completely excluded, rapid freezing in liquid nitrogen was performed prior to lyophilization to minimize ice crystal-induced artifacts. Therefore, SEM analysis was primarily used for comparative evaluation of relative pore morphology and network organization among different formulations rather than direct representation of the fully hydrated hydrogel architecture. Quantitative analysis revealed composition-dependent differences in pore size (Fig. 2C) and porosity (Fig. 2D). Consistent trends were observed in equilibrium swelling behavior and degradation profiles among different formulations (Figure S1A, B).

Chemical characterization was performed to confirm successful polymer modification and hydrogel network formation. As shown in the ^1H NMR spectra (Fig. 2E), compared with native HA, OHA exhibited a new signal at 4.9–5.2 ppm, corresponding to hemiacetal protons formed between introduced aldehyde groups and adjacent hydroxyl groups, confirming successful oxidation of HA [49]. Following dopamine conjugation, characteristic aromatic proton signals appeared at approximately 6.7 ppm, together with methylene proton signals at approximately 2.88 ppm, indicating successful grafting of dopamine onto the OHA backbone [41, 50]. In addition, SerMA displayed newly emerged resonance peaks at approximately 5.7 and 6.1 ppm, corresponding to vinyl protons (–CH = CH₂) of methacrylate groups, as well as a characteristic methyl proton signal at approximately 1.9 ppm, confirming successful methacrylation of sericin. FT-IR spectra (Fig. 2F) further verified the chemical modifications. Compared with HA, OHA exhibited a characteristic absorption band at approximately 1730 cm⁻¹, corresponding to aldehyde C = O stretching, indicating successful oxidation [49]. After dopamine conjugation, enhanced absorption around 1630 cm⁻¹ was observed, which was attributed to amide bond formation between OHA and dopamine. SerMA exhibited characteristic amide I and amide II bands at approximately 1630 cm⁻¹ and 1530 cm⁻¹, respectively, together with ester-related absorption associated with methacrylate modification. In the final OHA-DA/SerMA hydrogel, a characteristic absorption band at approximately 1655 cm⁻¹ was observed, which was partially attributed to C = N stretching vibrations, suggesting the formation of Schiff-base interactions between OHA-DA and SerMA. These spectral changes collectively confirmed successful synthesis of OHA-DA, methacrylation of sericin, and formation of the dual-crosslinked hydrogel network.

Rheological properties were assessed to characterize gelation kinetics and viscoelastic behavior of the hydrogels. Time-sweep measurements were performed at 1% strain and 1 Hz frequency. Following 405 nm light irradiation (30 mW/cm²) for 60 s, G′ rapidly increased and exceeded G″ within approximately 5 s. Prior to light irradiation, all formulations exhibited relatively low storage modulus (G′) and loss modulus (G″) values (Fig. 2H). Upon 405 nm light exposure, G′ rapidly increased and exceeded G″ within approximately 5 s, indicating rapid sol–gel transition and formation of a mechanically stable network. All formulations reached stable plateau G′ values within 60–80 s, with OHDSM-3 exhibiting the highest final G′ value. The hydrogels displayed pronounced shear-thinning behavior over a shear-rate range of 0.1–100 s⁻¹ (Fig. 2I), supporting their injectability. Strain-sweep analysis performed at 1 Hz frequency demonstrated stable viscoelastic behavior within the tested strain range (Fig. 2J). Frequency-sweep tests conducted at 1% strain further confirmed elastic-dominant behavior across a wide frequency range, with G′ consistently exceeding G″ (Fig. 2K). Step-strain cycling experiments alternating between 1% and 400% strain demonstrated rapid and reproducible recovery of G′ following repeated high-strain deformation, indicating robust self-healing capability of the dual-network hydrogel system (Fig. 2L).

Hydration and mechanical properties were further evaluated. All formulations reached hydration equilibrium within 24 h, with comparable equilibrium water uptake values (Fig. 2M). Uniaxial compression testing revealed nonlinear elastic stress–strain behavior for all hydrogels (Fig. 2O), with composition-dependent differences in compressive strength (Figure S1C). Wet-tissue adhesion was assessed using a lap-shear test. Representative stress–strain curves and quantitative adhesion strength measurements are shown in Fig. 2N and P, respectively, demonstrating composition-dependent adhesion performance. To further visually evaluate the adhesive capability of the hydrogel on biological tissues, qualitative wet-tissue adhesion experiments were performed using freshly harvested rat organs. The hydrogel remained firmly attached to multiple tissue surfaces under inverted conditions, confirming its robust wet-tissue adhesive performance (Figure S4).

Based on these physicochemical and mechanical characterizations, OHDSM-3 was selected for subsequent in vitro and in vivo experiments.

### Isolation, characterization, and cellular uptake of hypoxia-preconditioned ADSC-derived exosomes

**Fig. 3:**
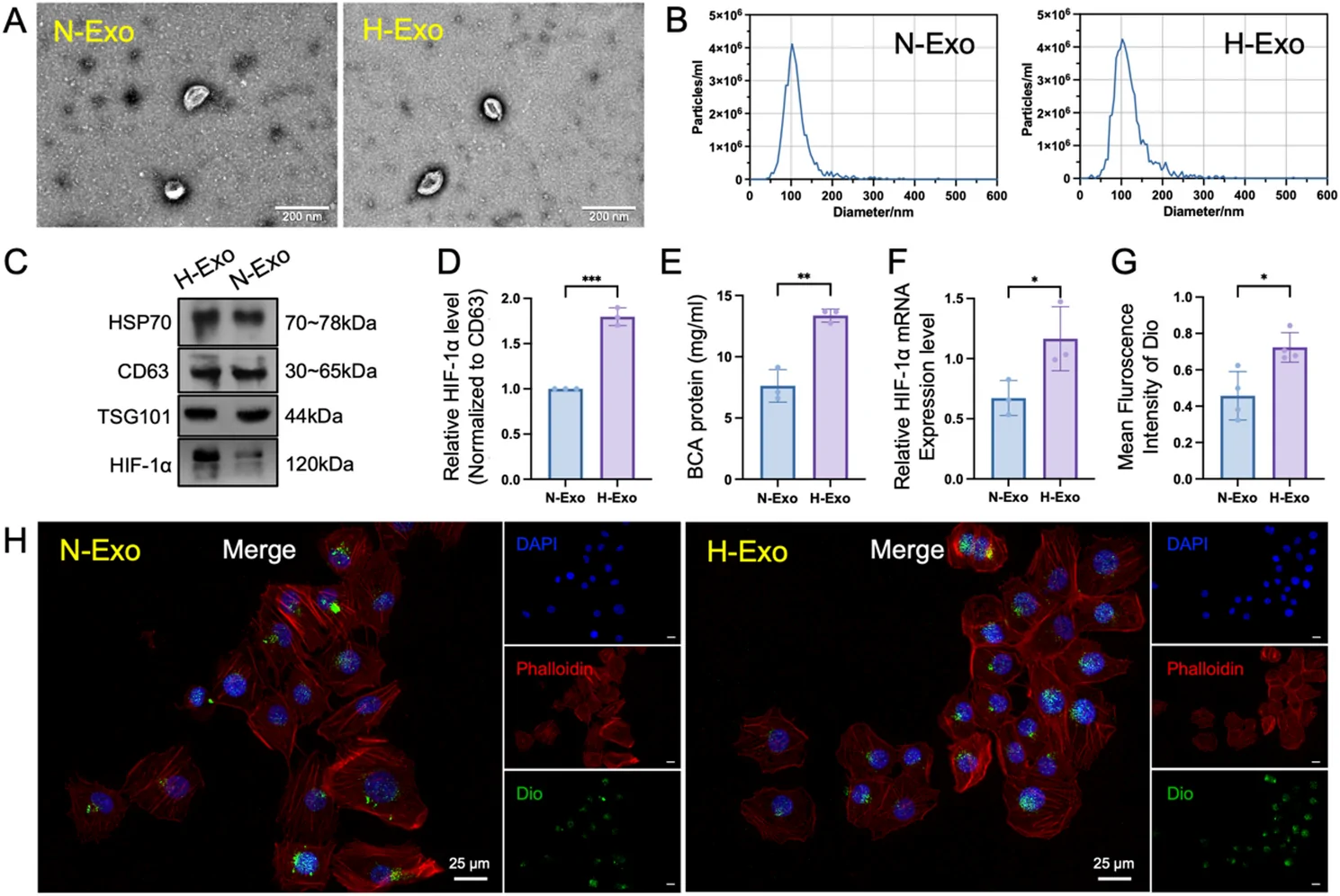
Comprehensive characterization of normoxia-derived exosomes (N-Exo) and hypoxia-preconditioned exosomes (H-Exo), including their physicochemical properties, molecular cargo enrichment, and cellular internalization behavior. (**A**) Transmission electron microscopy (TEM) images showing the typical cup-shaped morphology of N-Exo and H-Exo. (**B**) Nanoparticle tracking analysis (NTA) demonstrating comparable size distributions of N-Exo and H-Exo within the characteristic exosomal diameter range. (**C**) Western blot analysis confirming the presence of canonical exosomal markers (HSP70,CD63 and TSG101) and hypoxia-inducible factor-1α (HIF-1α) in N-Exo and H-Exo. (**D**) Densitometric quantification of exosomal HIF-1α protein levels normalized to CD63, indicating significant enrichment in H-Exo. (**E**) Total exosomal protein content quantified by BCA assay, showing increased cargo loading in H-Exo compared with N-Exo. (**F**) Relative HIF-1α mRNA expression levels in N-Exo and H-Exo measured by RT-qPCR. (**G**) Quantitative analysis of cellular uptake of DiO-labeled exosomes by endothelial cells, presented as mean fluorescence intensity. (**H**) Representative confocal microscopy images showing intracellular uptake of DiO-labeled N-Exo and H-Exo by endothelial cells. Actin cytoskeleton was stained with phalloidin (red), nuclei with DAPI (blue), and exosomes with DiO (green). Scale bars: 25 μm (merged images)

ADSC-derived exosomes were isolated from cells cultured under normoxic (N-Exos) or hypoxic (H-Exos) conditions using differential ultracentrifugation. The physicochemical characteristics of the isolated vesicles were first evaluated. Transmission electron microscopy (TEM) showed that both N-Exos and H-Exos displayed typical cup-shaped morphology with intact lipid bilayers (Fig. 3A). Nanoparticle tracking analysis (NTA) demonstrated comparable size distributions between the two groups, with particle diameters within the expected exosomal range (Fig. 3B).

Exosomal marker expression was assessed by western blotting. Both N-Exos and H-Exos expressed canonical exosomal markers CD63 and HSP70 (Fig. 3C). Notably, HIF-1α protein levels were markedly higher in H-Exos compared with N-Exos. Densitometric analysis normalized to CD63 confirmed a statistically significant increase in HIF-1α protein abundance in H-Exos (Fig. 3D). Quantification of total exosomal protein content by BCA assay showed significantly higher protein concentration in H-Exos than in N-Exos (13.36 ± 0.53 vs. 7.63 ± 1.33 mg/mL, *p* < 0.01; Fig. 3E). RT-qPCR analysis further demonstrated elevated HIF-1α mRNA levels in H-Exos (Fig. 3F).

To evaluate cellular uptake, DiO-labeled exosomes were incubated with endothelial cells and examined by confocal microscopy. Both N-Exos and H-Exos were internalized and predominantly localized in the perinuclear cytoplasm (Fig. 3H). Quantitative fluorescence intensity analysis revealed significantly higher intracellular fluorescence in cells treated with H-Exos compared with N-Exos (Fig. 3G).

These results demonstrate that hypoxic preconditioning does not alter exosomal morphology or size distribution but increases cargo content and enhances endothelial cellular uptake efficiency. H-Exos were therefore selected for subsequent hydrogel loading and functional evaluation.

### OHA-DA/SerMA hydrogel enables sustained release and prolonged local retention of H-Exos

**Fig. 4:**
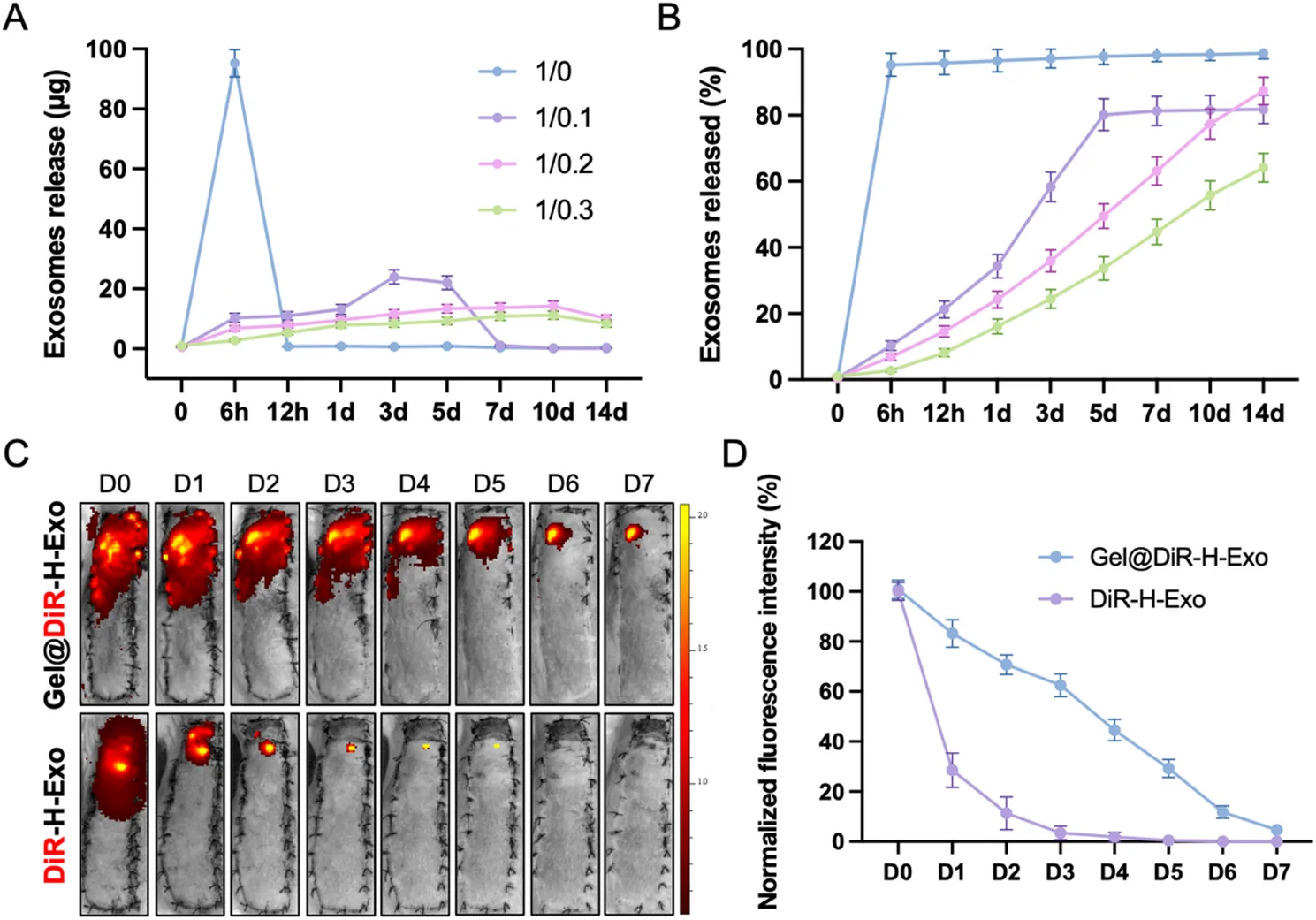
OHA-DA/SerMA hydrogel enables sustained release and prolonged local retention of H-Exos. (**A**) In vitro release profiles of exosomes from OHA-DA/SerMA hydrogels with different OHA-DA/SerMA ratios over a 14-day period. (**B**) Cumulative exosome release percentages of different hydrogel formulations, demonstrating sustained and composition-dependent release behavior. (**C**) Representative in vivo fluorescence images showing the retention of free DiR-labeled H-Exos (DiR-H-Exos) and hydrogel-loaded DiR-labeled H-Exos (Gel@DiR-H-Exos) within multiterritory perforator flaps from postoperative day 0 to day 7. (**D**) Quantitative analysis of fluorescence intensity within the flap region over time. Fluorescence signals were normalized to the initial signal intensity at day 0. Data are presented as mean ± SD (*n* = 3)

To evaluate the release behavior of H-Exos from the hydrogel system, exosome release profiles were assessed in vitro over a 14-day period. As shown in Fig. 4A, free exosomes (OHA-DA/SerMA = 1/0) exhibited rapid burst release, with nearly complete release occurring within the first 6 h. In contrast, hydrogels containing SerMA displayed markedly slower release kinetics. Among the tested formulations, increasing SerMA content progressively delayed exosome release. Consistently, cumulative release analysis (Fig. 4B) demonstrated a sustained and composition-dependent release pattern over 14 days, indicating that the dual-crosslinked hydrogel network effectively regulated exosome diffusion and prolonged release behavior.

To further investigate whether the hydrogel could improve exosome persistence in vivo, DiR-labeled H-Exos were tracked using IVIS imaging after local administration to multiterritory perforator flaps. As shown in Fig. 4C, fluorescence signals in the free DiR-H-Exo group rapidly decreased following administration and became nearly undetectable by postoperative day 4–5. In contrast, Gel@DiR-H-Exos maintained substantially stronger fluorescence signals throughout the observation period, with detectable signals persisting until postoperative day 7.

Quantitative ROI analysis further confirmed significantly enhanced local retention of exosomes in the Gel@DiR-H-Exo group compared with free exosomes (Fig. 4D). While fluorescence intensity in the free exosome group declined sharply within the first 48 h, hydrogel-mediated delivery markedly delayed signal loss and prolonged local exosome persistence within the choke zone.

Collectively, these findings demonstrate that the OHA-DA/SerMA hydrogel functions as an effective exosome delivery platform by enabling sustained release and prolonged local retention of H-Exos, thereby providing continuous bioavailability within the ischemic flap microenvironment.

### Gel@H-Exos reinvigorates endothelial bioactivity and angiogenic potential under oxidative stress

Oxidative stress is a primary pathological barrier in ischemic flaps, rapidly impairing endothelial function and regenerative capacity. To recapitulate this hostile microenvironment in vitro, HUVECs were exposed to hydrogen peroxide (H₂O₂; 600 µM) for 6 h to establish an oxidative injury model, which serves as a stringent platform to evaluate endothelial dysfunction characteristic of the choke zone [52]. 

**Fig. 5:**
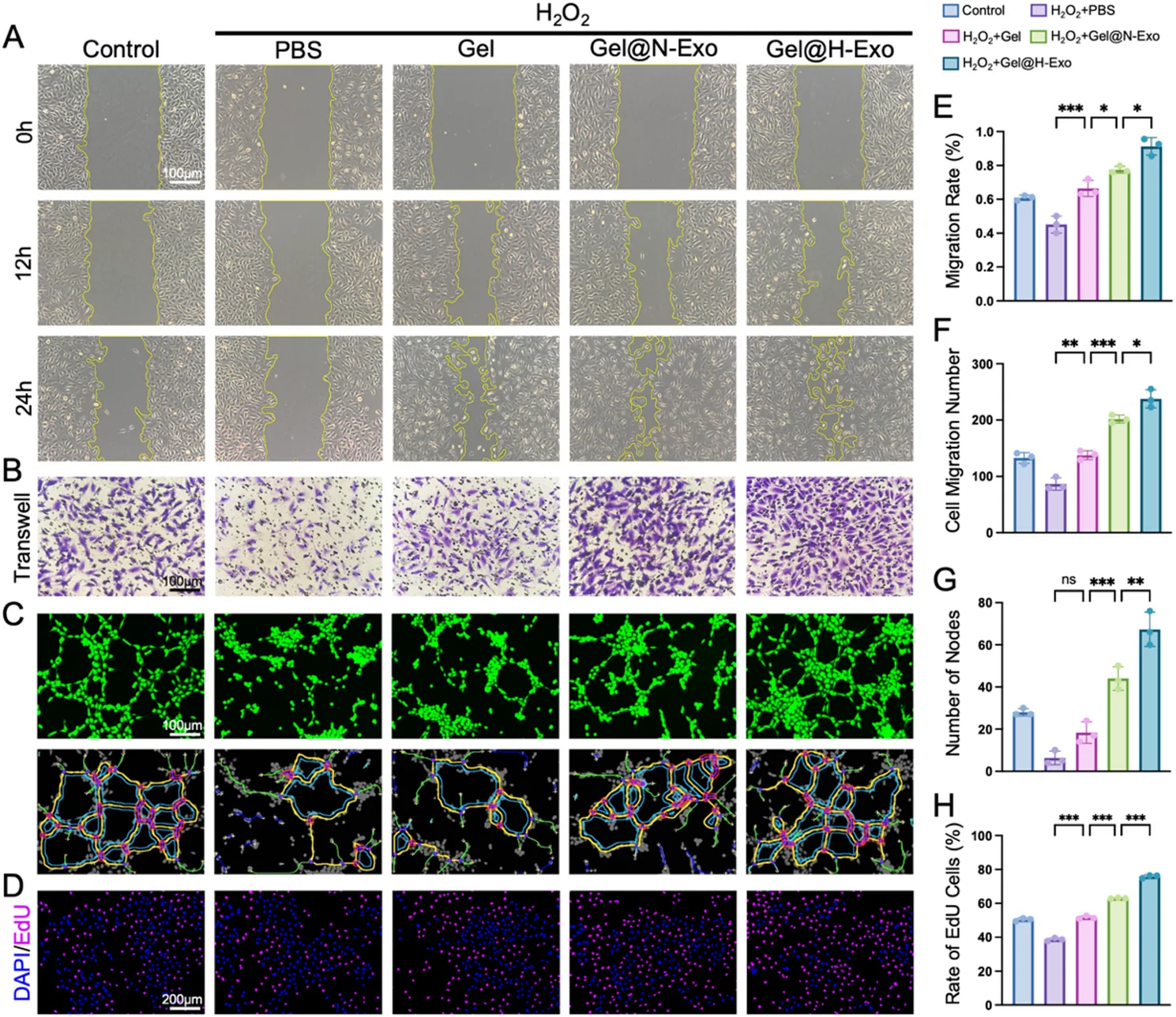
Gel@H-Exos restores endothelial migration, angiogenic activity, and proliferation under oxidative stress. (**A**) Representative scratch wound–healing assay images of endothelial cells at 0, 12, and 24 h under different treatments, including Control, H₂O₂ + PBS, H₂O₂ + Gel, H₂O₂ + Gel@N-Exo, and H₂O₂ + Gel@H-Exo. Scale bars: 100 μm. (**B**) Representative Transwell migration images of endothelial cells under the indicated conditions. Scale bars: 100 μm. (**C**) Representative images of Matrigel tube formation assays at 6 h, together with skeletonized images used for quantitative network analysis. Scale bars: 100 μm. (**D**) Representative EdU incorporation assay images showing proliferative activity of endothelial cells under oxidative stress. Scale bars: 200 μm. (**E**) Quantification of endothelial migration rate based on wound closure analysis. (**F**) Quantification of migrated cell number in the Transwell assay. (**G**) Quantification of angiogenic network connectivity expressed as the number of nodes in tube formation assays. (**H**) Quantification of endothelial proliferation expressed as the percentage of EdU-positive cells. Data are presented as mean ± SD. Statistical significance is indicated as *P < 0.05, **P < 0.01, ***P < 0.001; ns, not significant

Restoration of endothelial motility is a prerequisite for vascular repair. Scratch wound healing and Transwell migration assays were performed to assess migratory capacity under oxidative stress. As shown in Fig. 5A, B and H₂O₂ exposure markedly suppressed endothelial migration, resulting in delayed wound closure and reduced transmembrane movement. Treatment with the antioxidant hydrogel alone (Gel) or Gel@N-Exos partially alleviated this impairment. In contrast, **Gel@H-Exos** markedly enhanced migratory behavior, as evidenced by accelerated wound closure and increased numbers of migrated cells. Quantitative analysis confirmed that Gel@H-Exos significantly improved migration rate (Fig. 5E) and cell migration number (Fig. 5F) compared with other treatment groups.

In addition to migration, the ability to form organized capillary-like networks is a defining hallmark of angiogenic competence. This was evaluated using the Matrigel tube formation assay. Oxidative stress severely disrupted endothelial morphogenesis, leading to sparse and fragmented network structures (Fig. 5C). Notably, Gel@H-Exos treatment effectively rescued tubulogenic activity, enabling endothelial cells to assemble into dense and interconnected networks with enhanced structural complexity. Quantification of network connectivity demonstrated a significant increase in the number of nodes in the Gel@H-Exos group (Fig. 5G), indicating superior angiogenic organization relative to that of the other groups.

Finally, endothelial proliferative capacity was examined using the EdU incorporation assay. H₂O₂ challenge markedly reduced the proportion of proliferating cells, reflecting oxidative stress-induced cell cycle arrest (Fig. 5D). While the hydrogel alone exerted a modest protective effect, likely attributable to its intrinsic ROS-scavenging properties, incorporation of hypoxia-preconditioned exosomes (Gel@H-Exos) resulted in the most pronounced preservation of proliferative activity. Quantitative analysis revealed a significantly higher percentage of EdU-positive cells in the Gel@H-Exos group compared with that in the PBS group (Fig. 5H).

Collectively, these results demonstrate a synergistic therapeutic effect of the OHA-DA/SerMA hydrogel and hypoxia-preconditioned exosomes. The hydrogel component attenuates acute oxidative stress, thereby creating a permissive microenvironment, while sustained delivery of H-Exos actively restores endothelial migration, angiogenic organization, and proliferation. This functional reprogramming of endothelial cells under oxidative stress establishes a solid biological foundation for subsequent mechanistic investigations into pyroptosis inhibition.

### Gel@H-Exos attenuates oxidative stress, preserves mitochondrial function, and suppresses endothelial pyroptosis

**Fig. 6:**
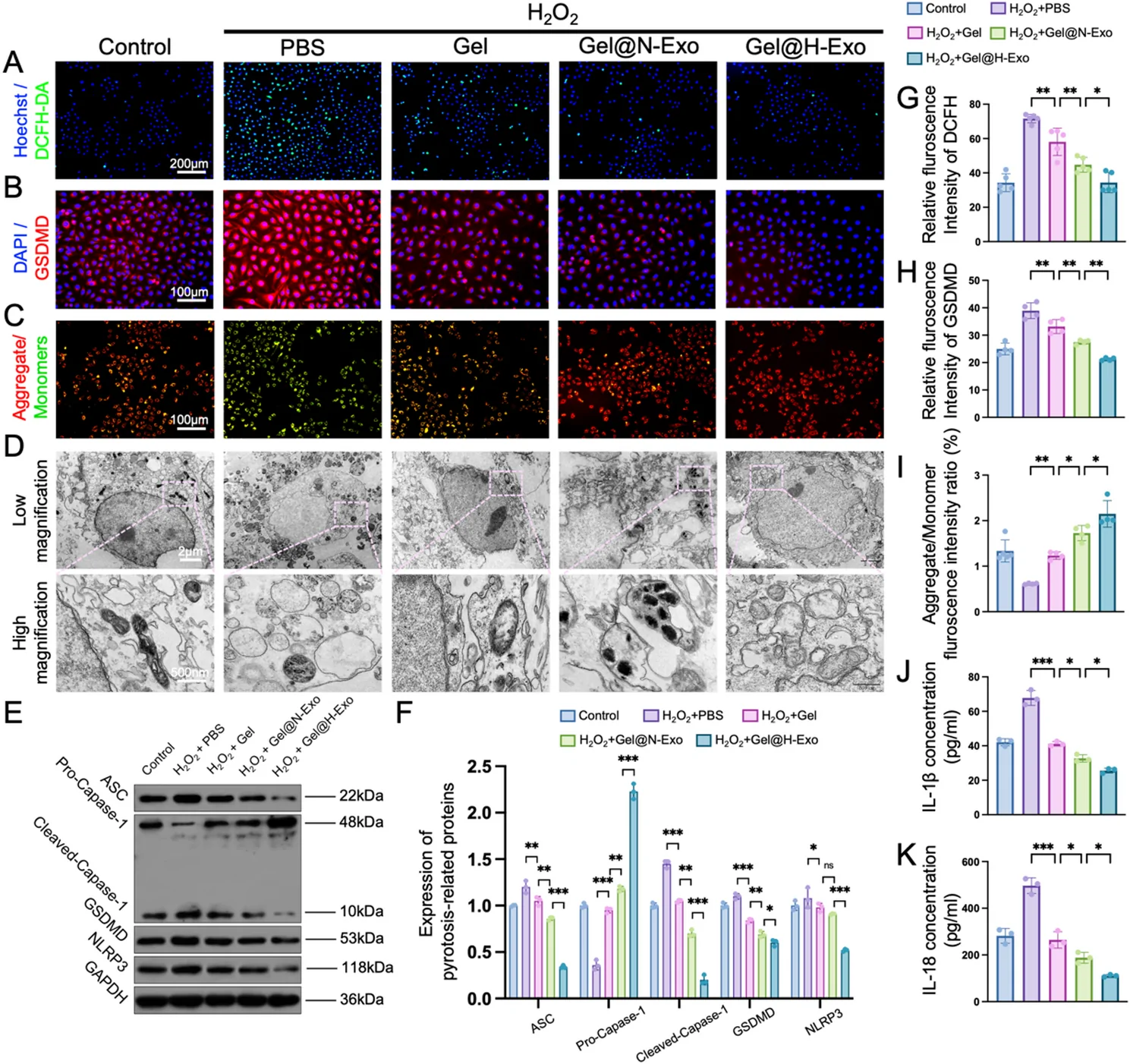
Gel@H-Exos alleviates oxidative stress, preserves mitochondrial integrity, and suppresses endothelial pyroptosis. (**A**) Intracellular reactive oxygen species (ROS) levels detected by DCFH-DA staining in endothelial cells under different treatments. Nuclei were counterstained with Hoechst (blue). Scale bar: 200 μm. (**B**) Immunofluorescence staining of GSDMD (red) in endothelial cells under oxidative stress. Nuclei were stained with DAPI (blue). Scale bar: 100 μm. (**C**) JC-1 staining showing changes in mitochondrial membrane potential (ΔΨm), with red fluorescence indicating JC-1 aggregates and green fluorescence indicating monomers. Scale bar: 100 μm. (**D**) Transmission electron microscopy (TEM) images showing ultrastructural alterations of endothelial cells under oxidative stress. Representative low-magnification images (upper panels) and corresponding high-magnification images (lower panels) highlight mitochondrial swelling, cristae disruption, and membrane integrity. Scale bars: 2 μm (low magnification) and 500 nm (high magnification). (**E**) Western blot analysis of pyroptosis-related proteins, including ASC, Pro-Caspase-1, Cleaved-Caspase-1, GSDMD, and NLRP3. (**F**) Densitometric quantification of pyroptosis-related protein expression normalized to GAPDH. (**G**) Quantitative analysis of intracellular ROS levels based on DCFH-DA fluorescence intensity. (**H**) Quantitative analysis of GSDMD fluorescence intensity. (**I**) Quantification of mitochondrial membrane potential expressed as the JC-1 aggregate-to-monomer fluorescence ratio. (**J**) Concentration of IL-1β in culture supernatants measured by ELISA. (**K**) Concentration of IL-18 in culture supernatants measured by ELISA. Data are presented as mean ± SD. Statistical significance is indicated as *P < 0.05, **P < 0.01, ***P < 0.001; ns, not significant

Intracellular reactive oxygen species (ROS) levels were first evaluated in HUVECs exposed to H₂O₂. Oxidative stimulation induced a marked increase in intracellular ROS fluorescence. As shown in Fig. 6A, Gel@H-Exos treatment significantly reduced ROS intensity compared with other treatment groups. Quantitative analysis confirmed a significant decrease in intracellular ROS levels in the Gel@H-Exos group (Fig. 6G).

Mitochondrial membrane potential (ΔΨm) was assessed using JC-1 staining. H₂O₂ exposure resulted in a shift from red fluorescent aggregates to green monomers, indicating mitochondrial depolarization (Fig. 6C). Gel@H-Exos treatment preserved ΔΨm, as reflected by an increased red/green fluorescence ratio (Fig. 6I). Transmission electron microscopy further revealed that mitochondria in H₂O₂-treated cells exhibited swelling and disrupted cristae, whereas cells treated with Gel@H-Exos maintained relatively intact mitochondrial ultrastructure (Fig. 6D).

Expression of pyroptosis-related proteins was subsequently examined. Immunofluorescence staining demonstrated increased GSDMD signal intensity in H₂O₂-treated cells, which was markedly reduced following Gel@H-Exos treatment (Fig. 6B). Quantification of mean fluorescence intensity confirmed this reduction (Fig. 6H). Western blot analysis showed that H₂O₂ stimulation upregulated NLRP3, ASC, cleaved caspase-1, and GSDMD expression (Fig. 6E). In contrast, Gel@H-Exos treatment significantly decreased the expression of these proteins. Densitometric analysis normalized to loading controls supported these findings (Fig. 6F).

To assess downstream inflammatory cytokine release, ELISAs were performed to quantify IL-1β and IL-18 levels. H₂O₂ exposure significantly increased secretion of both cytokines, whereas Gel@H-Exos treatment markedly reduced IL-1β (Fig. 6J) and IL-18 (Fig. 6K) levels.

These data indicate that Gel@H-Exos reduces oxidative stress, maintains mitochondrial integrity, and inhibits activation of the NLRP3 inflammasome signaling pathway in endothelial cells under oxidative conditions.

### Gel@H-Exos improves perfusion and survival of multiterritory perforator flaps in vivo

A rat multiterritory perforator flap model was established by preserving the deep circumflex iliac (DCI) artery as the sole vascular pedicle while ligating the thoracodorsal (TD) and intercostal (IC) vessels (Fig. 1C). This configuration generated a perfusion gradient across the flap, with distal territories dependent on collateral circulation through choke vessels. The flap was subdivided into a proximal anatomical zone, an intermediate choke zone (Choke I), and a distal potential zone (Choke II) [8, 53, 54].

Immediately after flap elevation, hydrogel precursors (Gel, Gel@N-Exos, or Gel@H-Exos) were applied in situ over the choke zones. Rapid gelation enabled stable coverage of the target region throughout the postoperative period.

**Fig. 7:**
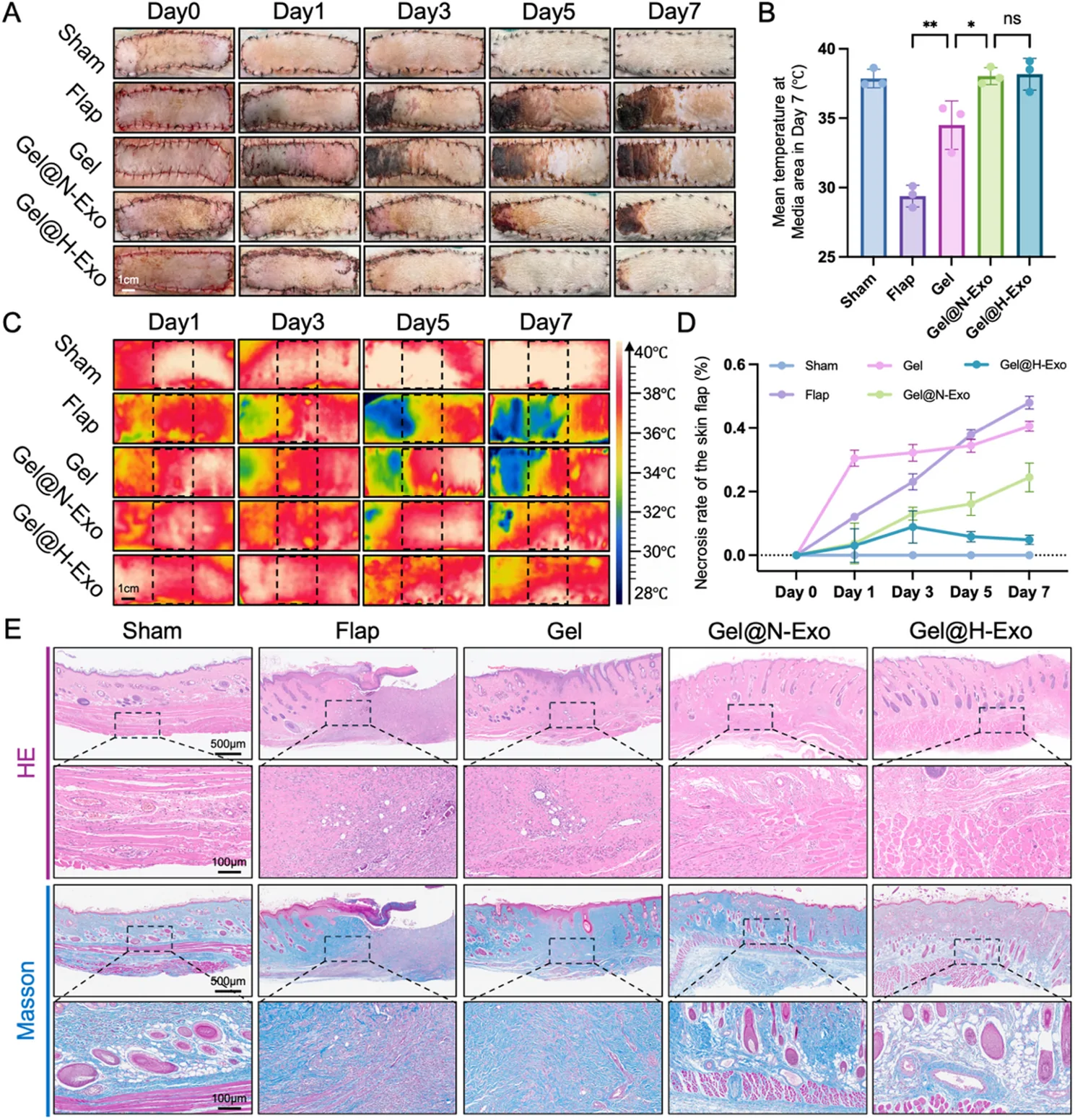
Gel@H-Exos improves perfusion and survival of multiterritory perforator flaps in vivo. (**A**) Representative gross images of multiterritory perforator flaps from different groups (Sham, Flap, Gel, Gel@N-Exo, and Gel@H-Exo) at postoperative Days 0, 1, 3, 5, and 7, showing the temporal progression of distal flap survival and necrosis. Scale bar: **1 cm**. (**B**) Quantitative analysis of mean surface temperature within the middle region of the flap (corresponding to the choke zone) at postoperative Day 7, measured by infrared thermography. (**C**) Representative infrared thermal images of flaps at postoperative Days 1, 3, 5, and 7, illustrating spatial and temporal changes in cutaneous perfusion. Dashed lines delineate the choke zone region. (**D**) Quantitative analysis of flap necrosis rate over time in different treatment groups. (**E**) Representative hematoxylin and eosin (H&E) and Masson’s trichrome staining of flap tissues harvested at postoperative Day 7. Low- and high-magnification images highlight epidermal integrity, inflammatory cell infiltration, collagen deposition, and overall tissue architecture. Scale bars: **500 μm** (low magnification) and **100 μm** (high magnification). Data are presented as mean ± SD. Statistical significance is indicated as *P* < 0.05, *P* < 0.01; ns, not significant

Macroscopic evaluation was performed to assess flap viability. As shown in Fig. 7A, all flaps appeared viable on postoperative Day 0. By Day 7, the Flap and Gel groups exhibited evident distal necrosis characterized by darkened tissue and clear demarcation lines. In contrast, the Gel@H-Exos group showed markedly reduced necrotic areas and improved preservation of distal tissue morphology. Quantitative analysis demonstrated a significantly lower necrotic area in the Gel@H-Exos group compared with the Flap and Gel@N-Exos groups (Fig. 7D).

Infrared thermography was used to assess cutaneous perfusion. Representative thermal images are shown in Fig. 7C. The Flap and Gel groups displayed persistently reduced surface temperatures in the choke zone, whereas Gel@H-Exos treatment resulted in higher thermal signals in this region. Quantitative analysis revealed a significantly increased mean temperature in the choke zone on postoperative Day 7 in the Gel@H-Exos group compared with the Flap group (Fig. 7B).

Histological evaluation was performed on postoperative Day 7. Hematoxylin and eosin (H&E) staining demonstrated extensive edema, inflammatory cell infiltration, and structural disruption in Flap and Gel-treated tissues (Fig. 7E). In contrast, Gel@H-Exos-treated flaps exhibited relatively preserved epidermal and dermal architecture with reduced inflammatory infiltration. Masson’s trichrome staining showed disorganized collagen deposition in control groups, whereas Gel@H-Exos-treated tissues displayed more organized collagen arrangement (Fig. 7E).

Histological examination of major organs revealed no observable pathological abnormalities in animals treated with Gel@H-Exos (Figure S2).

These findings indicate that Gel@H-Exos enhances microcirculatory perfusion and reduces distal necrosis in multiterritory perforator flaps.

### Gel@H-Exos promotes angiogenic remodeling and reduces endothelial pyroptosis in vivo

Flap tissues harvested from the choke zone (Region II) on postoperative day 7 were analyzed by immunohistochemistry (IHC) and immunofluorescence (IF).

**Fig. 8:**
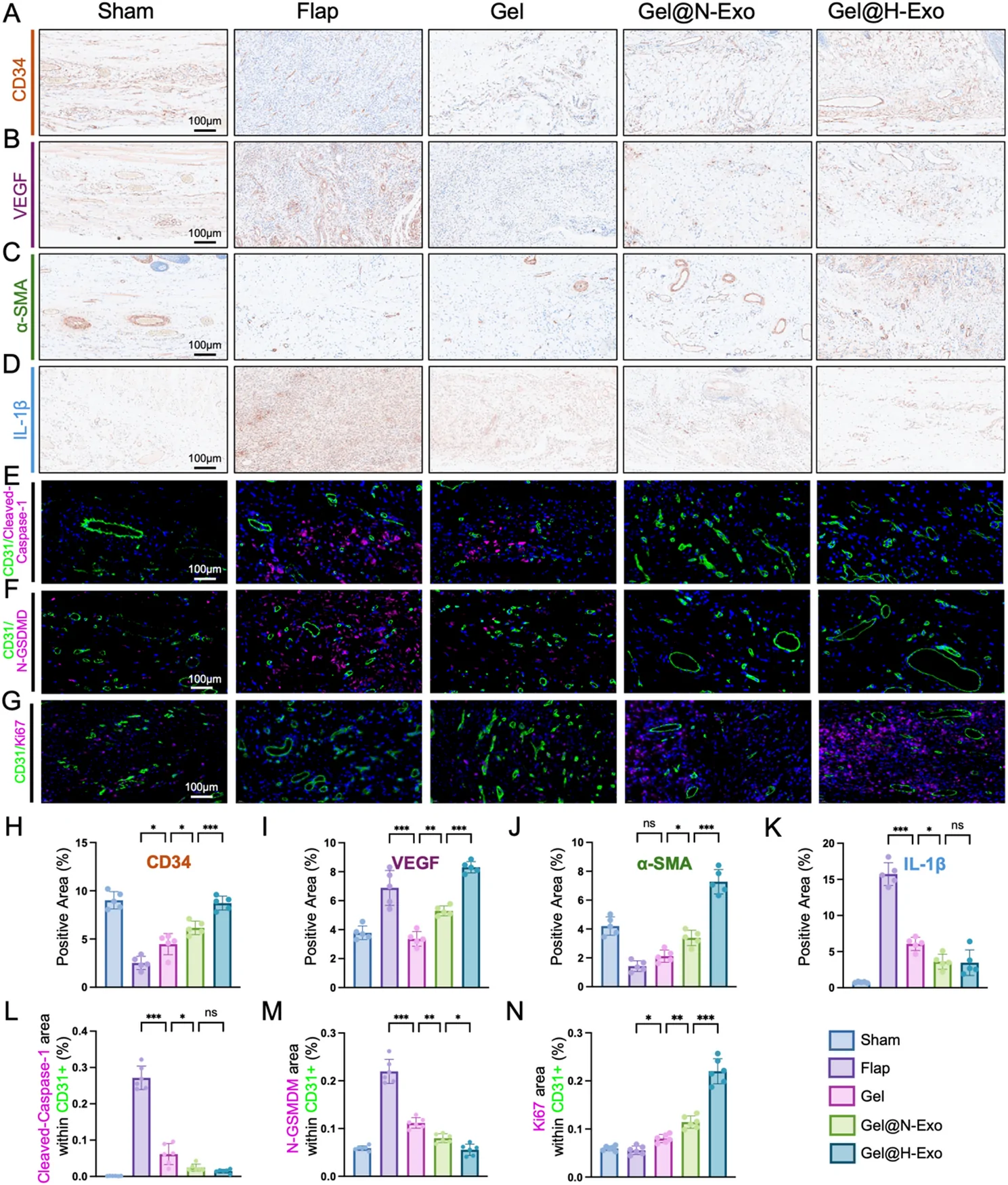
IHC and IF analyses of angiogenesis and endothelial pyroptosis in the choke zone on postoperative day 7. (**A**–**D**) Representative IHC staining images showing expression of angiogenesis- and inflammation-related markers, including CD34 (A, microvessel density), VEGF (B, pro-angiogenic signaling), α-SMA (C, perivascular smooth muscle coverage), and IL-1β (D, inflammasome-associated inflammatory response). Compared with the Flap and Gel groups, Gel@H-Exos–treated flaps exhibited increased CD34-, VEGF-, and α-SMA–positive staining, accompanied by markedly reduced IL-1β expression. Scale bars: 100 μm. (**E**, **F**) Representative IF co-staining images illustrating endothelial pyroptosis within flap tissues. CD31 (green) labels vascular endothelial cells, while cleaved Caspase-1 (E, magenta) and N-GSDMD (F, magenta) indicate inflammasome activation and pyroptotic execution, respectively. Gel@H-Exos treatment markedly reduced co-localization of pyroptotic markers within CD31⁺ vascular structures. Nuclei were counterstained with DAPI (blue). Scale bars: 100 μm. (**G**) Representative Ki67/CD31 double immunofluorescence staining images showing proliferating endothelial cells within flap tissues. CD31-positive endothelial cells are shown in green, Ki67-positive proliferating cells are shown in magenta, and nuclei were counterstained with DAPI (blue). Increased Ki67⁺/CD31⁺ co-localization was observed in the Gel@H-Exos group, indicating enhanced endothelial proliferation during angiogenic remodeling. Scale bars: 100 μm. (**H**–**K**) Quantitative analysis of CD34⁺ (**H**), VEGF⁺ (**I**), α-SMA⁺ (**J**), and IL-1β⁺ (K) tissue areas. (**L**, **M**) Quantification of cleaved Caspase-1⁺ (**L**) and N-GSDMD⁺ (**M**) signals within CD31⁺ vascular regions, reflecting the proportion of endothelial cells undergoing pyroptosis. (**N**) Quantitative analysis of Ki67⁺ area within CD31⁺ vascular regions, reflecting endothelial proliferative activity. Data are presented as mean ± SD. Statistical significance is indicated as *P < 0.05, **P < 0.01, ***P < 0.001; ns, not significant

Angiogenic remodeling and vascular maturation were first assessed. IHC staining revealed sparse CD34⁺ microvessels, reduced VEGF expression, and weak α-SMA⁺ perivascular coverage in the Flap and Gel groups (Fig. 8A–C). In comparison, Gel@H-Exos-treated flaps exhibited increased CD34⁺ capillary density, enhanced VEGF expression, and more continuous α-SMA staining surrounding vascular structures. Quantitative analysis further demonstrated significantly higher CD34⁺ (Fig. 8H), VEGF⁺ (Fig. 8I), and α-SMA⁺ (Fig. 8J) positive areas in the Gel@H-Exos group relative to other treatment groups. To further evaluate endothelial proliferation during angiogenic remodeling, Ki67/CD31 double immunofluorescence staining was performed. As shown in Fig. 8G, markedly increased Ki67⁺/CD31⁺ co-localization was observed in the Gel@H-Exos group compared with the Flap, Gel, and Gel@N-Exos groups. Quantitative analysis confirmed a significantly higher Ki67⁺ area within CD31⁺ vascular regions in the Gel@H-Exos group (Fig. 8N), further supporting enhanced endothelial proliferative activity within the ischemic choke zone.

Inflammatory signaling associated with pyroptosis was subsequently evaluated. IL-1β immunoreactivity was markedly elevated in the Flap and Gel groups (Fig. 8D), whereas Gel@H-Exos treatment significantly reduced IL-1β-positive staining within the choke zone. Quantitative analysis demonstrated a corresponding decrease in IL-1β-positive area in the Gel@H-Exos group (Fig. 8K).

To further assess endothelial pyroptosis directly, double immunofluorescence staining was performed for CD31 together with cleaved caspase-1 or N-GSDMD. Extensive co-localization of CD31 with cleaved caspase-1 (Fig. 8E) and N-GSDMD (Fig. 8F) was observed in the Flap and Gel groups. By contrast, Gel@H-Exos-treated flaps exhibited markedly reduced co-localization signals. Quantitative analysis of CD31⁺ endothelial cells co-expressing cleaved caspase-1 or N-GSDMD further confirmed significant reductions in cleaved caspase-1⁺ (Fig. 8L) and N-GSDMD⁺ (Fig. 8M) endothelial cells in the Gel@H-Exos group.

These data demonstrate that Gel@H-Exos enhances angiogenic remodeling and reduces endothelial pyroptosis-associated inflammation within the ischemic choke zone.

### Transcriptomic profiling reveals global transcriptional alterations induced by Gel@H-Exos

To characterize global transcriptional changes induced by Gel@H-Exos treatment, RNA sequencing (RNA-seq) was performed on flap tissues harvested from the choke zone (Region II) on postoperative day 7.

**Fig. 9:**
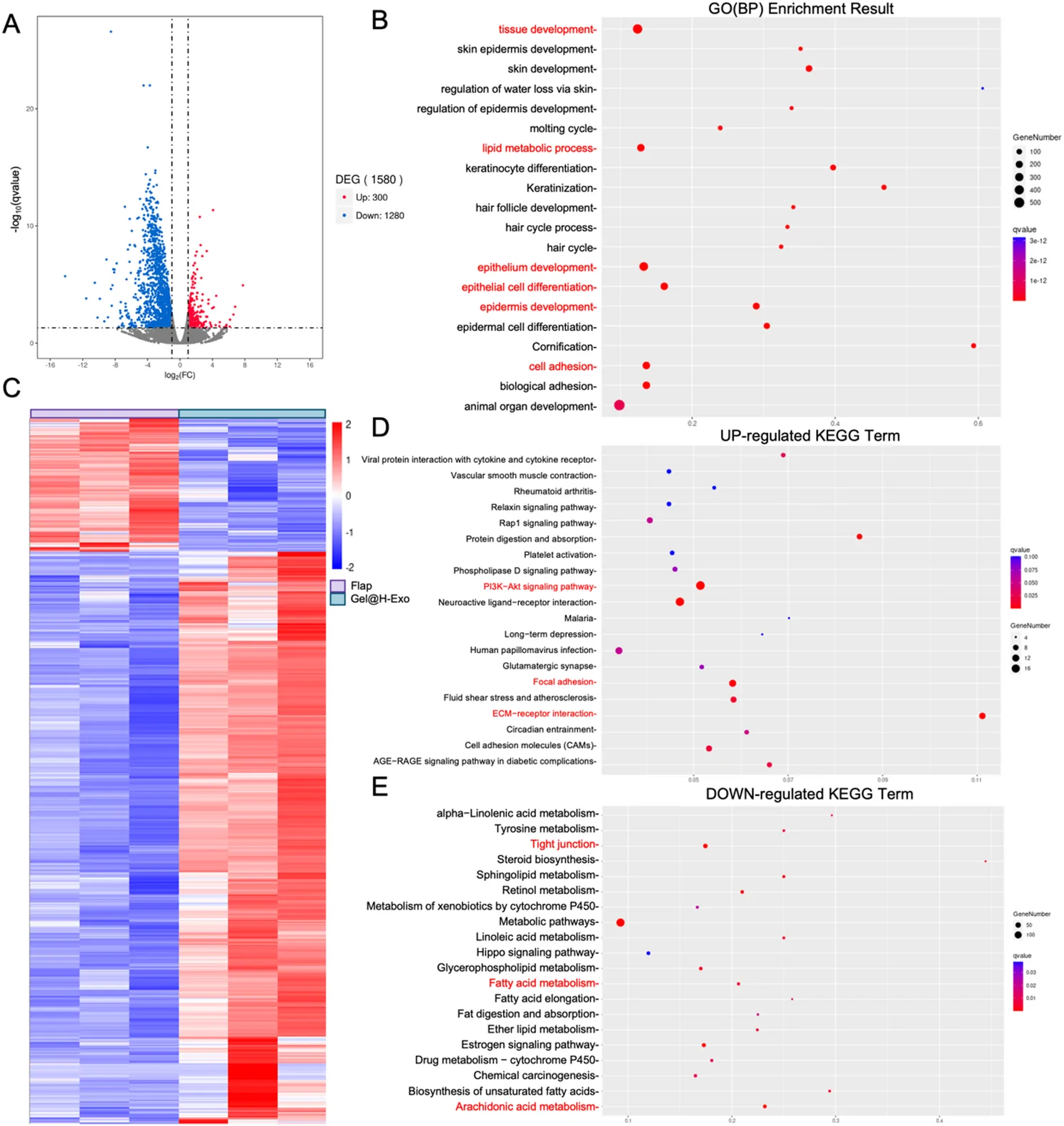
Transcriptomic profiling reveals coordinated tissue-level reprogramming induced by Gel@H-Exos. (**A**) Volcano plot showing differentially expressed genes (DEGs) between Gel@H-Exos–treated flaps and untreated control flaps. Each dot represents an individual gene. Red dots indicate significantly upregulated genes and blue dots indicate significantly downregulated genes, while gray dots represent genes that do not meet the differential expression criteria (|log₂ fold change| ≥ 1 and q < 0.05). Gel@H-Exos treatment induced extensive transcriptional remodeling, with a predominance of downregulated genes. (**B**) Gene Ontology (GO) biological process enrichment analysis of DEGs. Bubble plot displays representative enriched GO terms, with bubble size corresponding to the number of associated genes and color indicating enrichment significance (q value). Enriched processes are predominantly associated with tissue and epidermal development, epithelial cell differentiation, keratinocyte differentiation, and cell adhesion, reflecting enhanced structural regeneration and tissue remodeling. Lipid metabolic processes were also enriched, indicating concurrent modulation of metabolic pathways. (**C**) Hierarchical clustering heatmap of representative DEGs. Each column represents an individual sample and each row represents a gene. Gene expression levels are normalized and shown as Z-scores, with red and blue indicating relatively high and low expression levels, respectively. Samples cluster primarily according to treatment group, demonstrating high intra-group consistency and clear transcriptional segregation between Gel@H-Exos–treated and untreated flaps. (**D**) KEGG pathway enrichment analysis of upregulated genes in Gel@H-Exos–treated flaps. Significantly enriched pathways include PI3K–Akt signaling, ECM–receptor interaction, focal adhesion, and Rap1 signaling, which are closely related to endothelial survival, cell adhesion, and angiogenic remodeling. (**E**) KEGG pathway enrichment analysis of downregulated genes in Gel@H-Exos–treated flaps. Enriched pathways are mainly associated with lipid metabolic processes, including arachidonic acid metabolism, fatty acid metabolism, glycerophospholipid metabolism, and biosynthesis of unsaturated fatty acids, pathways known to contribute to inflammatory lipid mediator production and metabolic stress

Differential expression analysis identified substantial transcriptional changes between Gel@H-Exos–treated and untreated flaps. The volcano plot (Fig. 9A) shows numerous significantly upregulated and downregulated genes in response to Gel@H-Exos treatment. Hierarchical clustering and heatmap analysis demonstrated clear segregation between experimental groups with high intra-group consistency (Fig. 9C).

Gene Ontology (GO) biological process enrichment analysis revealed that upregulated differentially expressed genes (DEGs) were primarily associated with epidermis development, epithelial cell differentiation, cell adhesion, and extracellular matrix organization (Fig. 9B).

Kyoto Encyclopedia of Genes and Genomes (KEGG) pathway enrichment analysis showed significant enrichment of PI3K-Akt signaling, ECM-receptor interaction, and focal adhesion pathways among upregulated genes (Fig. 9D).

Conversely, downregulated pathways included arachidonic acid metabolism, fatty acid metabolism, and biosynthesis of unsaturated fatty acids (Fig. 9E).

These results demonstrate that Gel@H-Exos treatment induces coordinated transcriptional changes in ischemic flap tissues, involving pathways associated with tissue remodeling and inflammatory metabolism.

Although canonical pyroptosis- or inflammasome-related pathways were not identified among the top enriched GO or KEGG categories, several enriched pathways, including PI3K-Akt signaling, ECM-receptor interaction, and inflammatory metabolic regulation, are closely associated with endothelial survival, inflammatory responses, and microenvironment remodeling. Given that the RNA-seq analysis was performed using whole choke-zone tissues containing multiple cell populations, including endothelial cells, fibroblasts, inflammatory cells, and other stromal components, transcriptional signatures related to tissue repair and vascular remodeling may predominate over cell-specific pyroptotic signals in global enrichment analyses. Therefore, the transcriptomic findings were interpreted in conjunction with the protein-level, histological, and immunofluorescence data, which consistently demonstrated suppression of NLRP3 inflammasome activation and endothelial pyroptosis following Gel@H-Exos treatment.

## Discussion

In the present study, we demonstrate that sustained local delivery of hypoxia-preconditioned adipose-derived stem cell exosomes via a bioadhesive antioxidant hydrogel significantly enhances perfusion recovery and survival of multiterritory perforator flaps. This therapeutic effect is mechanistically associated with attenuation of oxidative stress, preservation of mitochondrial integrity, suppression of NLRP3 inflammasome activation, and reduction of endothelial pyroptosis both in vitro and in vivo. Transcriptomic profiling further suggests coordinated activation of regenerative pathways alongside repression of inflammatory metabolic programs within the ischemic choke zone, indicating that Gel@H-Exos induces broad tissue-level remodeling rather than isolated molecular changes.

Distal necrosis in multiterritory perforator flaps arises from sustained ischemia across adjacent angiosomes, particularly within the choke zone where perfusion depends on dynamic microvascular adaptation [15, 48, 52]. Under these conditions, excessive reactive oxygen species accumulation and mitochondrial dysfunction act as key upstream events that destabilize endothelial homeostasis [32, 55]. Although apoptosis and necrosis have traditionally been implicated in ischemic tissue injury, increasing evidence supports a central role for endothelial pyroptosis in amplifying inflammatory damage and disrupting microvascular continuity [9, 56]. Activation of the NLRP3 inflammasome triggers caspase-1–mediated cleavage of gasdermin D, leading to pore formation, membrane rupture, and release of IL-1β and IL-18, which further propagate local inflammation and impair vascular regeneration [10, 12, 47]. In our model, endothelial cells within the choke zone exhibited elevated expression of cleaved caspase-1 and N-GSDMD following ischemic challenge, suggesting involvement of pyroptotic execution in flap deterioration. Importantly, Gel@H-Exos markedly reduced these pyroptosis-associated signals while preserving endothelial structural integrity, suggesting that interruption of the oxidative stress–mitochondrial dysfunction–inflammasome cascade is a critical mechanism underlying improved flap survival.

The therapeutic strategy employed here integrates two complementary components: a catechol-functionalized antioxidant hydrogel and hypoxia-preconditioned exosomes. The OHA-DA/SerMA hydrogel provides strong wet-tissue adhesion and intrinsic reactive oxygen species–scavenging capacity, thereby mitigating acute oxidative burden within the ischemic niche [38, 44]. This redox modulation likely stabilizes mitochondrial membrane potential and limits inflammasome priming [29, 33, 57]. In parallel, hypoxia-preconditioned exosomes deliver bioactive cargos enriched under low-oxygen conditioning, including hypoxia-responsive signaling molecules, which enhance endothelial proliferation, migration, and angiogenic organization [29]. Compared with exosomes alone, incorporation into an adhesive hydrogel improves local retention and enables sustained release, addressing a major limitation of cell-free therapies in highly perfused or mechanically dynamic tissues [24]. To directly verify this delivery advantage, we further performed in vitro release and in vivo fluorescence retention studies. The results demonstrated that the OHA-DA/SerMA hydrogel enabled gradual exosome release over a 14-day period, whereas free exosomes exhibited rapid burst diffusion. Moreover, IVIS imaging revealed prolonged retention of Gel@H-Exos within the choke zone, with detectable fluorescence signals persisting for up to 7 days after flap surgery. In contrast, free exosomes were rapidly cleared from the implantation site. These findings provide direct evidence that hydrogel encapsulation enhances local exosome bioavailability, which may contribute to the superior therapeutic efficacy observed in flap regeneration and vascular remodeling. The coordinated reduction of ROS accumulation, preservation of mitochondrial morphology, suppression of inflammasome activation, and enhancement of vascular maturation suggest that effective repair of ischemic flap tissues requires both structural support and molecular regulation of the local microenvironment. Consistent with these findings, Gel@H-Exos treatment increased Ki67⁺/CD31⁺ endothelial signals within the choke zone, indicating enhanced endothelial proliferative activity during vascular remodeling. Together with the increased expression of CD34, VEGF, and α-SMA, these results suggest that Gel@H-Exos promotes both neovascular formation and vascular maturation in ischemic flap tissues.

Free hypoxia-preconditioned exosomes partially restored endothelial migration, angiogenic activity, proliferative capacity, and resistance to oxidative stress under H₂O₂ stimulation (Figure S3), confirming the intrinsic therapeutic potential of H-Exos. Compared with normoxic exosomes, H-Exos consistently exhibited stronger endothelial protective effects, in line with the enhanced bioactivity induced by hypoxic preconditioning. Nevertheless, hydrogel-mediated delivery produced more pronounced therapeutic benefits in vivo, which may be attributed to improved local retention and sustained exosome availability within ischemic tissues.

The present study further provides direct evidence supporting this delivery advantage. In vitro release analysis demonstrated that the OHA-DA/SerMA hydrogel enabled gradual exosome release over an extended period, whereas free exosomes exhibited rapid burst diffusion. Consistently, in vivo fluorescence imaging showed that hydrogel encapsulation markedly prolonged exosome retention within the choke zone after flap surgery. Unlike free exosomes, which dissipated rapidly after administration, Gel@H-Exos maintained detectable fluorescence signals for up to 7 days. These findings support the concept that prolonged local bioavailability contributes to the enhanced therapeutic efficacy observed in ischemic flap repair.

The functional advantages of this hydrogel system are likely related to the cooperative effects of its dual-crosslinked network. Dynamic Schiff-base interactions between OHA-DA and SerMA may contribute to rapid gelation, injectability, and reversible network remodeling, while photo-induced covalent crosslinking mediated by methacrylate groups enhances structural stability and limits premature exosome diffusion. In addition, catechol groups introduced through dopamine conjugation may strengthen wet-tissue adhesion and improve local retention under mechanically dynamic ischemic conditions. Although the individual contribution of each crosslinking mechanism was not independently evaluated, the observed rheological behavior, tissue adhesion, and sustained-release characteristics collectively support the functional integration of dynamic and covalent crosslinking within the hydrogel network.

Transcriptomic analysis provides additional insight into the systemic impact of Gel@H-Exos treatment. Upregulation of pathways related to extracellular matrix organization, cell adhesion, PI3K-Akt signaling, and focal adhesion suggests reinforcement of structural remodeling and survival signaling within the regenerating tissue. Concurrent downregulation of arachidonic acid and fatty acid metabolic pathways indicates attenuation of pro-inflammatory lipid mediator production, consistent with reduced IL-1β expression and diminished endothelial pyroptosis observed histologically [58]. Although canonical pyroptosis-related pathways were not identified as independent enrichment categories in the GO or KEGG analyses, this observation should be interpreted with caution. Bulk RNA sequencing was performed using whole choke-zone tissues, which comprise diverse cellular populations, including endothelial cells, fibroblasts, immune cells, and other stromal components. Consequently, global enrichment analyses are more likely to capture dominant biological processes such as extracellular matrix remodeling, angiogenesis, tissue repair, and metabolic reprogramming, whereas endothelial pyroptosis represents a relatively cell-specific pathological event. Therefore, the absence of direct pyroptosis enrichment does not exclude its biological relevance. Instead, the transcriptomic findings should be integrated with the WB, ELISA, and IF results, all of which consistently demonstrated reduced NLRP3 inflammasome activation, decreased Caspase-1/GSDMD signaling, and diminished IL-1β production following Gel@H-Exos treatment. These genome-wide changes align with the observed improvements in perfusion, vascular maturation, and tissue architecture, supporting the concept that effective flap salvage depends on coordinated modulation of angiogenesis and inflammatory restraint.

Compared with previously reported strategies for ischemic flap salvage, the present approach emphasizes simultaneous regulation of oxidative stress, inflammatory signaling, and vascular remodeling [15, 53, 59]. Conventional pharmacological antioxidants primarily target reactive oxygen species without directly addressing downstream inflammasome activation or endothelial functional impairment [32]. Similarly, administration of stem cell–derived exosomes alone has shown promise in promoting angiogenesis and reducing inflammation; however, rapid diffusion, limited retention within ischemic tissues, and insufficient modulation of the hostile microenvironment often constrain therapeutic efficacy [24, 57]. In this context, the integration of an adhesive antioxidant hydrogel with hypoxia-preconditioned exosomes offers a combinatorial framework in which structural stabilization of the ischemic niche and sustained molecular delivery occur concurrently. Rather than functioning as a passive carrier, the hydrogel contributes intrinsic redox-regulating capacity, while hypoxia-conditioned exosomes reinforce pro-survival and pro-angiogenic signaling. This dual-level intervention appears particularly advantageous in the choke zone, where microvascular adaptation must occur within a narrow temporal window to prevent irreversible tissue loss.

Notably, the present study highlights endothelial pyroptosis as a modifiable pathological node in flap ischemia. While prior investigations have linked NLRP3 inflammasome activation to vascular dysfunction in diabetic wounds, atherosclerosis, and myocardial ischemia, its direct involvement in multiterritory perforator flap failure has been less clearly defined. By demonstrating co-localization of endothelial markers with cleaved caspase-1 and N-GSDMD in vivo, and showing that targeted intervention attenuates these signals, our findings extend the conceptual framework of flap pathophysiology beyond perfusion deficits alone. These results suggest that microvascular collapse in the choke zone is not merely a hemodynamic consequence of reduced inflow but also an active inflammatory process driven by pyroptotic execution. Therapeutic strategies aimed at preserving endothelial viability may therefore have broader implications for reconstructive procedures characterized by ischemia-reperfusion stress.

Despite these encouraging findings, several limitations should be acknowledged. First, although the rat multiterritory perforator flap model reproduces key features of choke-zone ischemia, interspecies differences may limit direct extrapolation to human reconstructive surgery. Second, while hypoxia-preconditioned exosomes demonstrated enhanced therapeutic efficacy, the specific bioactive cargos responsible for inflammasome suppression and angiogenic enhancement were not comprehensively identified. In addition, the transcriptomic analysis was performed at the whole-tissue level and therefore could not fully resolve cell-type-specific transcriptional responses. Future studies employing single-cell transcriptomics or spatial transcriptomic approaches may provide a more comprehensive understanding of how Gel@H-Exos regulates endothelial pyroptosis within the complex ischemic microenvironment. Future studies employing proteomic and microRNA profiling, as well as gain- and loss-of-function approaches, will be necessary to delineate precise molecular mediators. Third, the present analysis focused primarily on early to mid-term outcomes. Long-term evaluation of hydrogel degradation kinetics, sustained microvascular remodeling, and potential immunogenicity will be required to fully assess translational feasibility. In addition, although in vitro release profiling and in vivo retention imaging demonstrated that the dual-crosslinked hydrogel effectively prolonged local exosome retention, the individual contribution of dynamic Schiff-base interactions and photo-induced covalent crosslinking to sustained release behavior and therapeutic efficacy was not independently quantified in the present study. Future studies employing single-network control systems or selectively disrupted crosslinking architectures may further clarify the relative contribution of dynamic and covalent crosslinking to hydrogel stability, exosome retention, and therapeutic performance.

In summary, the present study demonstrates that an adhesive antioxidant hydrogel enabling sustained delivery of hypoxia-preconditioned exosomes effectively attenuates endothelial pyroptosis, promotes angiogenic remodeling, and enhances survival of multiterritory perforator flaps. By targeting both structural and inflammatory determinants of microvascular failure, this strategy provides a mechanistically informed platform for improving outcomes in ischemia-compromised reconstructive procedures.

## Conclusion

We engineered a bioadhesive and antioxidant OHA-DA/SerMA hydrogel as a localized niche for the sustained delivery of hypoxia-preconditioned exosomes. By integrating stable wet-tissue adhesion with microenvironment-regulating capacity, the Gel@H-Exos system effectively stabilizes the ischemic and oxidative milieu at the dynamic flap interface, overcoming key physiological barriers that limit the efficacy of conventional exosome-based therapies.

Our results indicate that Gel@H-Exos mitigates endothelial dysfunction by attenuating oxidative stress-driven inflammasome activation and pyroptosis. Preservation of mitochondrial homeostasis and suppression of caspase-1/GSDMD signaling collectively support functional vascular remodeling and rescue of the critical choke zone in a clinically relevant multiterritory perforator flap model. At the tissue level, a coordinated shift toward a pro-regenerative and anti-inflammatory state occurred, characterized by repression of inflammatory lipid metabolism and activation of structural and angiogenic programs.

Collectively, this study highlights that microenvironment-targeted reprogramming of endothelial fate, rather than isolated pro-angiogenic stimulation, is an effective strategy for restoring perfusion in ischemic tissues. Future studies should focus on elucidating cargo-specific mechanisms underlying exosome-mediated inflammasome regulation, optimizing dosing and delivery parameters, and validating long-term safety and efficacy in large-animal or clinically relevant models. Together, these efforts will further advance the translational potential of the Gel@H-Exos platform for complex reconstructive surgery and a broad spectrum of ischemia-related disorders.

## Supplementary Information

Below is the link to the electronic supplementary material.

Supplementary Material 1

## Data Availability

The data in this study are available upon reasonable request.

## Abbreviations

^1^H NMR: ^1^H nuclear magnetic resonance
ADSC: Adipose-derived stem cell
BCA: Bicinchoninic acid
DCI: Deep circumflex iliac
DEG: Differentially expressed gene
EDC: 1-ethyl-3-(3-dimethylaminopropyl) carbodiimide
Exos: Exosomes
FT-IR: Fourier-transform infrared
GO: Gene Ontology
H&E: Hematoxylin and eosin
H-Exos: Hypoxia-preconditioned adipose-derived stem cell exosomes
HA: Hyaluronic acid
HRP: Horseradish peroxidase
HUVEC: Human umbilical vein endothelial cell
IC: Posterior intercostal
KEGG: Kyoto Encyclopedia of Genes and Genomes
LAP: Lithium phenyl-2,4,6-trimethylbenzoylphosphinate
N-Exos: Normoxic exosomes
NHS: N-hydroxysuccinimide
NTA: nanoparticle tracking analysis
OHA: Oxidized hyaluronic acid
OHA-DA: Oxidized hyaluronic acid-dopamine
PBS: Phosphate-buffered saline
RNA-seq: RNA sequencing
ROS: Reactive oxygen species
SerMA: Sericin methacrylate
TD: Thoracodorsal
