# Supplementary material for: A bioadhesive antioxidant dual-crosslinked hydrogel mitigates endothelial pyroptosis to enhance ischemic flap survival
Source: J Nanobiotechnology. 2026 Jul 3;24:906. doi: 10.1186/s12951-026-04767-7 (PMC13617720; doi:10.1186/s12951-026-04767-7)
Supplement: Supplementary file 1 [file 12951_2026_4767_MOESM1_ESM.docx]

**Supporting Information**

**Figure S1. Supplementary physicochemical characterization of OHA-DA/SerMA hydrogels.** (A) In vitro mass loss profiles of OHA-DA/SerMA hydrogels with different compositions over time, demonstrating composition-dependent degradation behavior. (B) Equilibrium swelling ratios of hydrogels with varying OHA-DA/SerMA ratios, reflecting differences in network density and water uptake capacity. (C) Compressive strength of hydrogels with different compositions, indicating tunable mechanical properties regulated by hydrogel formulation. (D) In vitro degradation rates of hydrogels at a defined time point, showing a gradual reduction in degradation with increasing OHA-DA content.

**Figure S2. In vivo biosafety evaluation of OHA-DA/SerMA hydrogel implantation.** Representative hematoxylin and eosin (H&E) staining images of major organs, including heart, liver, spleen, lung, and kidney, harvested after hydrogel implantation. No apparent histopathological abnormalities, inflammatory infiltration, or tissue damage were observed, indicating good in vivo biocompatibility of the OHA-DA/SerMA hydrogel.

**Figure S3. Free hypoxia-preconditioned exosomes partially restore endothelial function under oxidative stress.** (A) Representative scratch wound-healing assay images of HUVECs at 0, 12, and 24 h under different treatments, including Control, H₂O₂ + PBS, H₂O₂ + N-Exo, and H₂O₂ + H-Exo. Scale bars: 100 μm. (B) Representative Matrigel tube formation images and corresponding skeletonized images used for quantitative network analysis. Scale bars: 100 μm. (C) Representative Transwell migration images showing endothelial migratory capacity under different treatments. Scale bars: 50 μm. (D) Representative EdU incorporation assay images showing proliferative activity of HUVECs. EdU-positive nuclei are shown in magenta and total nuclei were counterstained with DAPI (blue). Scale bars: 200 μm. (E) Representative DCFH-DA fluorescence images showing intracellular ROS levels under oxidative stress. Nuclei were counterstained with Hoechst (blue). Scale bars: 200 μm. (F–J) Quantitative analysis of migration rate (F), tube formation node number (G), migrated cell number in the Transwell assay (H), percentage of EdU-positive cells (I), and intracellular ROS fluorescence intensity (J). Data are presented as mean ± SD. Statistical significance is indicated as *P < 0.05, **P < 0.01, ***P < 0.001.

**Figure S4. Qualitative evaluation of wet-tissue adhesion of OHA-DA/SerMA hydrogel.** Representative photographs showing stable adhesion of the hydrogel to freshly harvested rat organs under inverted conditions, demonstrating strong wet-tissue adhesive capability.
